# DNA-Interactive and Damage Study with *meso*-Tetra(2-thienyl)porphyrins Coordinated with Polypyridyl Pd(II) and Pt(II) Complexes

**DOI:** 10.3390/molecules28135217

**Published:** 2023-07-05

**Authors:** Bernardo Almeida Iglesias, Níckolas Pippi Peranzoni, Sophia Iwersen Faria, Luana Belo Trentin, André Passaglia Schuch, Otávio Augusto Chaves, Renan Ribeiro Bertoloni, Sofia Nikolaou, Kleber Thiago de Oliveira

**Affiliations:** 1 Bioinorganic and Porphyrinoids Materials Laboratory, Department of Chemistry, Federal University of Santa Maria (UFSM), Santa Maria 97105-900, RS, Brazil; 2 Laboratory of Photobiology, Department of Biochemistry and Molecular Biology, Federal University of Santa Maria (UFSM), Santa Maria 97105-900, RS, Brazil; nickolas.pippiperanzoni@gmail.com (N.P.P.); sossofaria10@gmail.com (S.I.F.); luanabelotrentin@gmail.com (L.B.T.); schuchap@gmail.com (A.P.S.); 3 CQC-IMS, Department of Chemistry, University of Coimbra, Rua Larga, 3004-535 Coimbra, Portugal; otavioaugustochaves@gmail.com; 4 Laboratory of Biological Activity and Supramolecular Chemical of Coordination Compounds (LABiQSC2), Department of Chemistry, FFCLRP, University of São Paulo, Ribeirão Preto 14040-901, SP, Brazil; renanrbert@usp.br (R.R.B.); sofia@ffclrp.usp.br (S.N.); 5 Department of Chemistry, Federal University of São Carlos, Rod. Washington Luiz, km 235, São Carlos 13565-905, SP, Brazil

**Keywords:** porphyrins, thienyl-porphyrins, Pt(II) and Pd(II) complexes, DNA, photobiology

## Abstract

We report the DNA-binding properties of three porphyrins with peripheral thienyl substituents (**TThPor**, **PdTThPor** and **PtTThPor**). The binding capacity of each porphyrin with DNA was determined by UV-Vis and steady-state fluorescence emission spectroscopy combined with molecular docking calculations. The results suggest that the interaction of these compounds probably occurs via secondary interactions via external grooves (minor grooves) around the DNA macromolecule. Moreover, porphyrins containing peripheral Pd(II) or Pt(II) complexes (**PdTThPor** and **PtTThPor**) were able to promote photo-damage in the DNA.

## 1. Introduction

Porphyrins belong to a large family of tetrapyrrole macrocycles. These heterocycles are present in nature and play a key role in the metabolism of organisms and plants. Generally, they can be found in metalloenzymes and used as inorganic dyes, photosensitizers and catalysts for chemical and biological purposes [1,2,3,4,5]. Based on the chemical and physical properties of porphyrins, these compounds are often studied as photosensitizers in photodynamic therapy (PDT) and photodynamic inactivation (PDI) treatments [6,7]. PDT is a non-invasive targeted approach that incorporates a photosensitizer (PS), which can bio-accumulate in malignant tumor cells. Thus, the combination of PS, light sources and molecular oxygen can induce microorganism inactivation and tumor cell destruction. In PDT or PDI treatments, for example, photosensitizers generate singlet oxygen (^1^O_2_) by energy transfer (Type II) or radical species (such as •OH and O_2_^•−^) by electron transfer (Type I) using appropriate light dosage [8,9].

Notably, tetra(thienyl)porphyrin derivatives (**TThPor**) and their metallo-complexes are a relatively underexplored class of aryl-substituted porphyrins that deserve investigation due to their photophysical, photochemical and chemical properties [10]. Different from tetra(phenyl)porphyrin derivatives (**TPPs**), the **TThPor** compounds differ not only by the molecular formula but also by how the aryl rings are conjugated with the porphyrin core structure, with the thienyl moieties almost in a coplanar conformation in relation to the core structure. This very particular structural property may also result in different biological interactions [10]. Therefore, the use of square-planar complexes of Pd(II) and Pt(II) on the periphery of porphyrins is suitable due to their favorable stereochemistry and possible lability of ligands during the interaction.

Palladium(II) and platinum(II) complexes can interact with DNA through supramolecular properties or even through the formation of covalent bonds in nucleobases, thus hindering replication of the resulting DNA. This biomacromolecule exists in many possible conformations, including the most common forms such as the A-DNA, B-DNA and Z-DNA polymorphs, with the B-DNA and Z-DNA forms being the most commonly observed in most organisms. In general, oxidative damage to DNA leads to mutations. Although guanine is present with cytosine, dihydroguanine (8-oxoG), which is the most frequent type of oxidative damage to this nucleobase, can cause improper pairing with adenine through a conformational change, which is a route for mutations [11,12]. In recent years, several studies have reported DNA interactions with this type of complex [13,14,15,16,17,18]. Concerning inducing cancer cell death, developing compounds that target and damage DNA transcription and replication may represent an effective antitumor strategy.

**Figure 1:**
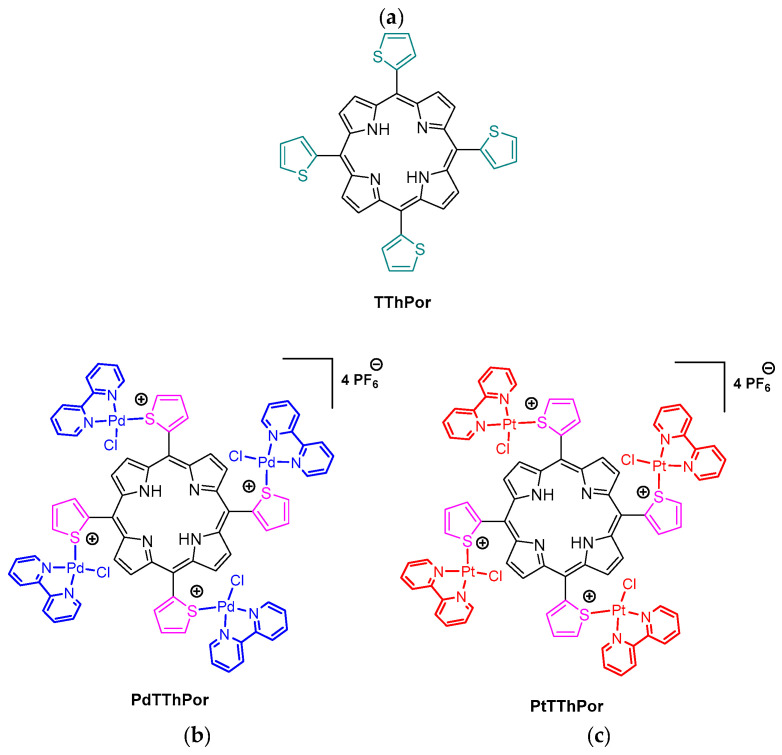
Representative structures of tetra(2-thienyl)porphyrins (**a**) free-base **TThPor**, (**b**) palladium(II) **PdTThPor** and (**c**) platinum(II) **PtTThPor** complexes.

Based on the photophysical properties and biological importance of thienyl-porphyrins and considering the window of opportunities for biological studies with these compounds, in this study we report preliminary studies on the interaction of tetrathienyl porphyrins with peripheral Pd(II) or Pt(II) complexes (**PdTThPor** and **PtTThPor**, Figure 1) and biomolecules. The interaction of the porphyrins with DNA was evaluated by multiple spectroscopic techniques (UV-Vis, circular dichroism and steady-state fluorescence) and viscosity analysis, combined with molecular docking calculations. Additionally, DNA breakage damage by capillary electrophoresis technique and photo-oxidation studies by UV-Vis analysis were also performed.

## 2. Results

### 2.1. Thienyl-Porphyrins

The free-base *meso*-tetra(2-thienyl)porphyrin **TThPor** was previously synthesized and fully characterized as described in the literature [10]. The *meso*-tetra(2-thienyl)porphyrin with Pd(II) and Pt(II) peripheral complexes **PdTThPor** and **PtTThPor** were also prepared and fully characterized as described by Tisoco and co-workers [19].

### 2.2. DNA-Binding Assays

#### 2.2.1. Binding Properties of DNA by UV-Vis Analysis

**Figure 2:**
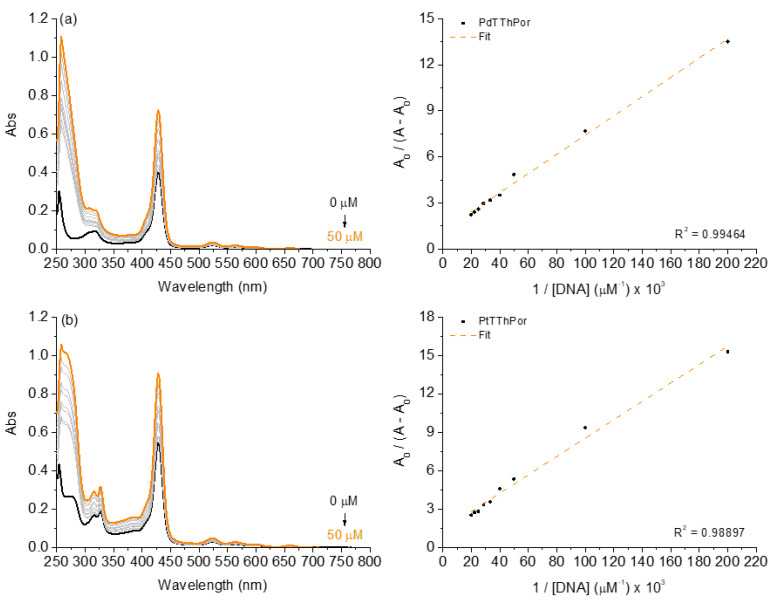
UV-Vis spectra of the (**a**) **PdTThPor** and (**b**) **PtTThPor** upon successive additions of CT-DNA concentrations (0 to 50 µM) in a DMSO(5%)/Tris-HCl pH 7.4 mixture buffered solution. Graph plots of A_0_/(A − A_0_) versus 1/[CT-DNA].

Aiming at evaluating the interaction between calf-thymus (CT-DNA) and the porphyrins **TThPor**, **PdTThPor** or **PtTThPor**, absorption analyses were carried out. The UV-Vis spectra for the thienyl porphyrins in the absence and presence of successive additions of CT-DNA concentrations are shown in Figure 2, and the DNA-binding properties are listed in Table 1. All UV-Vis spectra of porphyrin **TThPor** are presented in the Supplementary Information section (Figure S1).

Observing the absorption analyses, the successive additions of DNA to Pd(II) and Pt(II) compound solutions caused hyperchromic effects at the Soret and Q bands without a red or blue shift, indicating that the porphyrins can interact with DNA, probably via secondary interactions by external grooves around the DNA macromolecule. As derivatives containing peripheral complexes of Pd(II) and Pt(II) are positively charged (cationic), they have potential cationic-anionic electrostatic binding properties with DNA phosphate units, thus demonstrating a great interaction with nucleic acids, which is in agreement with several previous studies that reported the activity of positively charged tetrapyrrolic macrocycles [20,21,22,23].

In this way, the binding constant (*K*_b_) values were determined and are in the order of 10^4^ M^−1^ (Table 1), indicating that both porphyrins interact with CT-DNA and that the presence of peripheral complexes can interfere with the binding affinity of the DNA structure. Thermodynamic analysis via Gibbs free energy by ΔG° values (Table 1) indicated that all compounds interact spontaneously with DNA, thus reinforcing the results observed by *K*_b_ values. In the next section, the possibilities of interaction between these tetrathienyl porphyrins in terms of intercalation or via grooves are also investigated by steady-state fluorescence emission techniques.

#### 2.2.2. Competitive Binding Assays with DNA by Steady-State Fluorescence Emission

**Figure 3:**
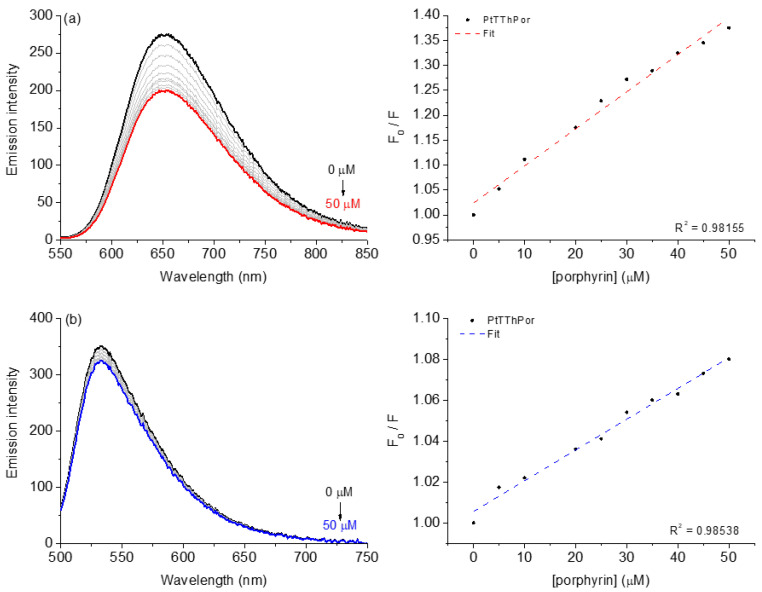

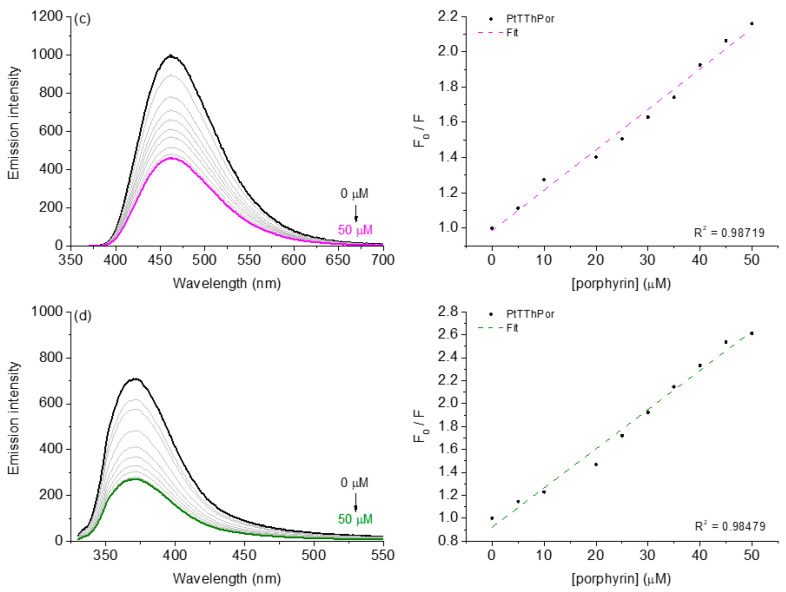
Steady-state fluorescence emission spectra for (**a**) EB:DNA, (**b**) AO:DNA, (**c**) DAPI:DNA and (**d**) MG:DNA without and in the presence of porphyrin **PtTThPor** in a DMSO(5%)/Tris-HCl pH 7.4 mixture buffered solution. Graph plots show the F_0_/F versus [porphyrin derivative].

The steady-state fluorescence emission analysis involving the competition assays for the binding between porphyrins and DNA:dye adducts was performed using porphyrin **PtTThPor** (Figure 3). The fluorescence Stern-Volmer (*K*_SV_), bimolecular quenching rate (*k*_q_), binding (*K*_b_) and ΔG° parameters for DNA:dye:porphyrin are listed in Table 1. All fluorescence emission spectra of porphyrins **TThPor** and **PdTThPor** with DNA:dye adducts are presented in the Supplementary Information section (Figures S2–S9).

As an example, the fluorescence emission spectra for ethidium bromide (EB) bound to DNA (fluorescence emission at 652 nm when excited at 510 nm) in the absence and presence of Pt(II) porphyrin **PtTThPor** are depicted in Figure 3a. When the porphyrin derivatives were added to DNA pre-treated with EB dye (general intercalator dye), the **PtTThPor** induced a decrease in the fluorescence intensity of the EB:DNA adduct, indicating a displacement of EB from DNA, which can be assigned as a viable competition between ethidium bromide dye and porphyrin for DNA strands. Additionally, the same behavior was observed for the other studied derivatives.

For acridine orange (AO) competition assays, the steady-state fluorescence emission spectra for AO bound to DNA (fluorescence emission at 534 nm when excited at 480 nm) in the absence and presence of porphyrin **PtTThPor** are depicted in Figure 3b. In this case, when porphyrins were added to DNA pre-treated with AO dye, the **PtTThPor** induced a slight decrease in the fluorescence intensity of the AO:DNA adduct, indicating a little displacement of AO from DNA, and the corresponding *K*_SV_, *k*_q_ and *K*_b_ constant values in the presence of the studied porphyrins. These values are listed in Table 1.

To evaluate whether the possible interaction of thienyl porphyrins occurs via the groove site, the minor and major grooves’ dyes: 4′,6-diamidino-2-phenylindole (DAPI) and methyl green (MG), respectively, were used for the steady-state fluorescence emission quenching assays (Figure 3c,d). In independent experiments, it was possible to observe a significant fluorescence quenching of DAPI:DNA and MG:DNA upon successive additions of porphyrins. Comparing both *K*_SV_ and *K*_b_ values for competitive binding assays into intercalator dyes EB:DNA and AO:DNA adducts, it can be inferred that there is a significant variation in the fluorescence quenching constants, mainly in the presence of the studied porphyrins (Table 1). Overall, the *K*_SV_ and *K*_b_ data variation (~10^4^ M^−1^) can be attributed to a preference for porphyrin interaction by the external grooves and not only by an intercalation phenomenon, agreeing with the CD, viscosity and molecular docking calculations as described in the next sections as well as in the literature [24,25].

Moreover, the *k*_q_~10^12^ M^−1^s^−1^ values for the porphyrins in DAPI and MG solutions are higher than the diffusion rate constant according to literature [26], thus indicating a ground-state interaction between porphyrins and DNA nuleobases, probably by a static mechanism).

**Table 1:** DNA-binding properties of porphyrins **TThPor**, **PdTThPor** and **PtTThPor** by UV-Vis and steady-state fluorescence emission analysis.

	**UV-Vis Analysis**				
**Porphyrin**	***H* (%) ^a^**	**Δλ (nm) ^b^**	***K*_b_ (×10^4^; M^−1^) ^c^**	**ΔG° (kcal mol ^−1^) ^d^**	
**TThPor**	63.0	0.0	0.92 ± 0.09	−5.40	
**PdTThPor**	44.5	0.0	1.59 ± 0.13	−5.75	
**PtTThPor**	40.0	0.0	1.39 ± 0.21	−5.65	
	**Steady-State Fluorescence Emission Analysis**				
	**EB:DNA**				
	***Q* (%) ^e^**	***K*_SV_ (×10^3^; M^−1^) ^f^**	***k*_q_ (×10^11^; M^−1^s^−1^) ^g^**	***K*_b_ (×10^3^; M^−1^) ^h^**	**ΔG° (kcal mol^−1^) ^d^**
**TThPor**	20.0	4.86 ± 0.04	2.11 ± 0.08	7.44 ± 0.47	−5.30
**PdTThPor**	27.0	6.14 ± 0.08	2.67 ± 0.15	14.3 ± 0.23	−5.65
**PtTThPor**	28.0	7.43 ± 0.01	3.23 ± 0.02	11.1 ± 0.18	−5.50
	**AO:DNA**				
	***Q* (%) ^e^**	***K*_SV_ (×10^3^; M^−1^) ^f^**	***k*_q_ (×10^12^; M^−1^s^−1^) ^i^**	***K*_b_ (×10^3^; M^−1^) ^h^**	**ΔG° (kcal mol^−1^) ^d^**
**TThPor**	23.0	5.27 ± 0.01	3.10 ± 0.02	5.35 ± 0.55	−5.10
**PdTThPor**	8.0	1.72 ± 0.01	1.01 ± 0.02	2.01 ± 0.83	−4.50
**PtTThPor**	7.5	1.50 ± 0.02	0.88 ± 0.04	1.63 ± 0.79	−4.40
	**DAPI:DNA**				
	***Q* (%) ^e^**	***K*_SV_ (×10^4^; M^−1^) ^f^**	***k*_q_ (×10^12^; M^−1^s^−1^) ^j^**	***K*_b_ (×10^4^; M^−1^) ^h^**	**ΔG° (kcal mol^−1^) ^d^**
**TThPor**	54.0	2.31 ± 0.05	10.5 ± 0.10	1.42 ± 0.13	−5.65
**PdTThPor**	36.0	8.98 ± 0.02	40.8 ± 0.04	0.96 ± 0.37	−5.45
**PtTThPor**	54.0	2.29 ± 0.03	10.4 ± 0.06	2.36 ± 0.14	−5.95
	**MG:DNA**				
	***Q* (%) ^e^**	***K*_SV_ (×10^4^; M^−1^) ^f^**	***k*_q_ (×10^12^; M^−1^s^−1^) ^k^**	***K*_b_ (×10^4^; M^−1^) ^h^**	**ΔG° (kcal mol^−1^) ^d^**
**TThPor**	38.5	1.25 ± 0.02	4.46 ± 0.04	3.58 ± 0.28	−6.20
**PdTThPor**	63.0	3.50 ± 0.02	12.5 ± 0.04	3.74 ± 0.03	−6.25
**PtTThPor**	62.0	3.42 ± 0.04	12.2 ± 0.08	2.77 ± 0.17	−6.05

^a^ *H*(%) = (A_0_ − A)/A × 100%; ^b^ Red-shift; ^c^ Binding constant by Benesi-Hidelbrandt equation; ^d^ Determined by Gibbs free-energy equation; ^e^ *Q*(%) = (F_0_ − F)/F × 100%; ^f^ Determined by Stern-Volmer quenching constant; ^g^ Determined by the *K*_SV_/τ_0_ ratio, where τ_0_ = 23 ns (EB:DNA) [27]; ^h^ Determined by the modified Stern-Volmer equation; ^i^ Determined by the *K*_SV_/τ_0_ ratio, where τ_0_ = 1.70 ns (AO:DNA) [28]; ^j^ Determined by the *K*_SV_/τ_0_ ratio, where τ_0_ = 2.20 ns (DAPI:DNA) [29]; ^k^ Determined by the *K*_SV_/τ_0_ ratio, where τ_0_ = 2.80 ns (MG:DNA) [30].

#### 2.2.3. Viscosity Measurements with DNA and Porphyrins

**Figure 4:**
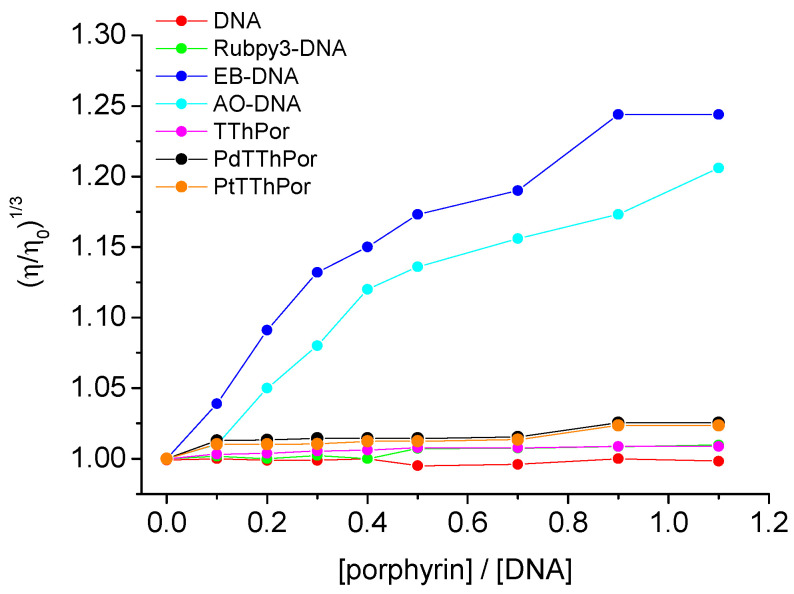
Viscosity assays in solution in a DMSO(5%)/Tris-HCl pH 7.4 mixture buffered solution with [porphyrin]/[DNA] ratios.

It is known that viscosity assays are sensitive to changes in DNA structure. As a complementary technique, viscosity analysis can be considered an efficient method to determine the possible intercalation or non-intercalation of compounds into DNA nucleobases [31]. The results of viscosity measurements of all porphyrins, **TThPor**, **PdTThPor** and **PtTThPor** are shown in Figure 4. The DNA viscosity remains almost unchanged upon the addition of porphyrin derivatives, with an increase in the ratio [porphyrin]/[CT-DNA]. These results indicate that tetra-thienyl porphyrins are not performing intercalation between the DNA bases, and they corroborate the steady-state fluorescence measurements in the presence of EB, DAPI, AO or MG (which probably bind to minor/major grooves). Despite the relative planarity of thienyl porphyrin derivatives, this is an expected result since these derivatives still have a steric volume that is not favorable to promoting an efficient intercalation phenomenon.

#### 2.2.4. Circular Dichroism (CD) Analysis with DNA

**Figure 5:**
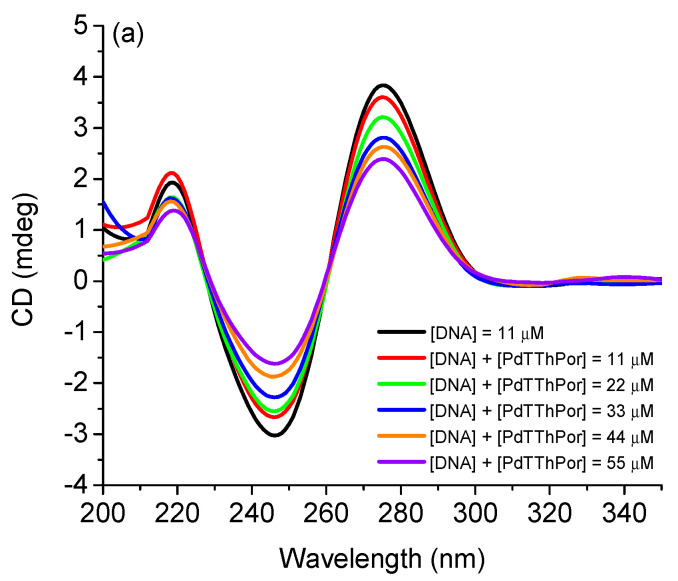

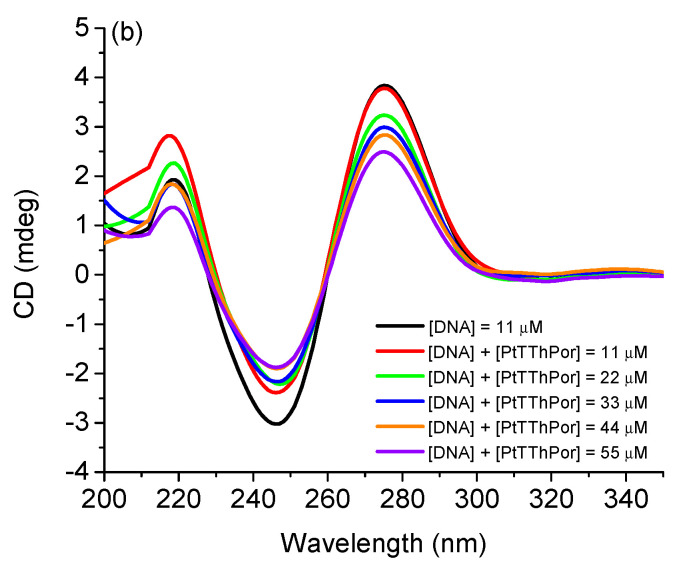
CD spectra for DNA solution in Tris-HCl buffer (pH = 7.4, 1% DMSO) before and after successive additions of porphyrins (**a**) **PdTThPor** and (**b**) **PtTThPor**.

The CD spectra illustrated in Figure 5 reveal that the tetra-cationic Pd(II) and Pt(II) porphyrins **PdTThPor** and **PtTThPor** interact with DNA since their addition causes a hypochromic effect in the bands present at 219, 246 and 275 nm, thus indicating an alteration in the DNA strands. In the CD spectra, the helicity characteristic of right-handed B-form DNA is represented by a negative band at 246 nm, while base stacking is identified by the positive band at 275 nm (Figure 5a,b). The CD spectra of free-base porphyrin **TThPor** are listed in the Supplementary Information section (Figure S10).

The first transition occurs in the band referring to amides, that is, the peptide bonds of DNA, while the transitions at 246 and 275 nm are attributed to aromatic amino acids [32,33]. Therefore, these last two bands strongly indicate an interaction of the DNA base pairs and porphyrins, since the addition of compounds directly affects the DNA structure, especially in the aromatic regions, altering the molar absorptivity at these wavelengths of circularly polarized light due to the drastic conformational changes in the overall structure [34,35]. This same behavior has already been observed for similar porphyrin derivatives containing peripheral platinum(II) complexes [36].

#### 2.2.5. Molecular Docking Analysis with DNA

Molecular docking is a useful approach to offering a molecular-level explanation of the binding capacity of small compounds to DNA [25]. Thus, in silico calculations via molecular docking were carried out to suggest the main intermolecular forces responsible for the binding process between the tetra-thienyl porphyrins and the DNA double-strand, as well as the corresponding binding site, i.e., minor or major groove and the key nucleobases. The docking score value (dimensionless) for DNA:porphyrins inside the most possible binding sites is shown in Table 2.

In the interaction between DNA and studied porphyrins, the highest docking score value was obtained for the minor groove, e.g., docking scores for DNA:**TThPor** of 69.0 and 44.5 in the minor and major grooves, respectively, suggesting that the porphyrins bind preferentially in the minor groove of DNA [37], agreeing with the experimental data reported above (dye displacement studies). Data from literature also indicated the minor groove of DNA strands as the main region for other porphyrins, including Ni(II)-[tetra-*N*-methyl-pyridyl]porphyrin [38], Mn(III)-bis-aqua-*meso*-tetrakis(4-*N*-methylpyridiniumyl)porphyrin [39], free-base and Zn(II)-*meso*-tetra(ruthenated)porphyrins [23].

**Figure 6:**
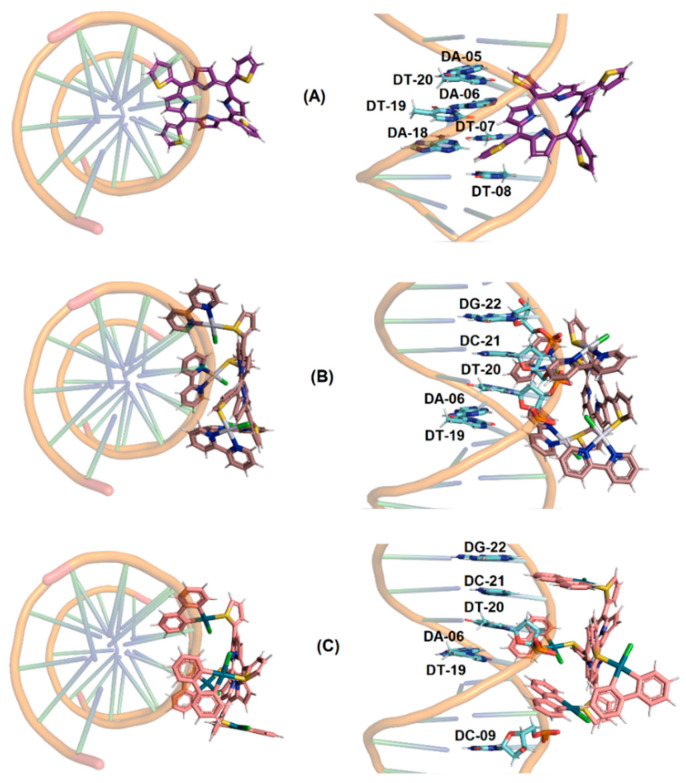
Best docking pose (top and front of representation) of the interaction (**A**) DNA:**TThPor**, (**B**) DNA:**PtTThPor** and (**C**) DNA:**PdTThPor** in the minor groove. Selected nitrogenated bases, **TThPor**, **PtTThPor** and **PdTThPor** are in stick representation in cyan, purple, brown and beige, respectively. Elements’ color: hydrogen: white; oxygen: red; nitrogen: dark blue; sulfur: yellow; chloro: green, Pt(II): silver; and Pd(II): dark green.

The best docking pose for the interaction between DNA:porphyrins in a minor groove is depicted in Figure 6. Molecular docking results suggested that the peripheral groups connected in the *meso* position of the porphyrin structure are interacting with the nucleobases, and the porphyrin core is more accessible to the aqueous medium than buried inside the DNA strands. Van der Waals interactions are the main forces responsible for the interaction between DNA and porphyrins, relating mainly with adenine and thymine nucleobases in the minor groove (see Supplementary Information section—Table S1). Finally, in silico calculations did not detect the possibility of intercalation between the porphyrins and DNA strands, corroborating the experimental viscometry data (see Section 2.2.3).

### 2.3. DNA Photo-Oxidation and Damage

#### 2.3.1. DNA Photo-Oxidation by Absorption Analysis

Aiming at evaluating the photo-oxidation processes between DNA and the studied porphyrins **TThPor**, **PdTThPor** and **PtTThPor**, UV-Vis absorption analyses were carried out in the presence of white-light irradiation conditions (irradiance of 50 mW cm^−2^ and a total light dosage of 90 J cm^−2^) at 298.15 K. All UV-Vis spectra of compounds are presented in the Supplementary Information section (Figures S11–S13).

As previously reported by Tisoco and co-workers, tetra-thienyl porphyrins containing Pd(II) and Pt(II) polypyridyl complexes generate reactive oxygen species (ROS) under light irradiation and can cause photo-damage to biomolecules such as serum albumins (in this case, HSA) [19]. The DNA photo-oxidation parameters in the absence and presence of selected porphyrins at fixed concentrations are shown in Table 3. It is possible to notice that the derivatives containing the peripheral complexes of Pd(II) and Pt(II) provide a decay of the DNA absorbance peak at 260 nm as the solution is irradiated with a white LED source. This fact agrees with the possibility of these derivatives to photo-oxidize biomolecules, and this is proven in the next tests of DNA damage by electrophoresis technique (see next section).

#### 2.3.2. DNA Oxidative Damage by Electrophoresis

Analyzing the gels, it is possible to observe the formation of DNA lesions when exposing the free-base **TThPor** to white-light conditions (see Supplementary Information section—Figure S14), mainly at 10 µM. These results, however, do not differ significantly from the control (*p* > 0.05). In dark conditions, there is no induction of damage by the tetra-thienyl porphyrin.

**Figure 7:**
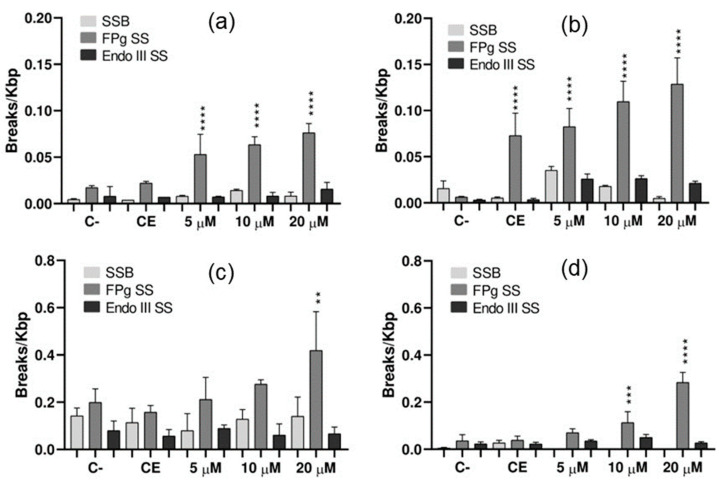
Quantification of DNA lesions generated by porphyrins (**a**) **PtTThPor** (dark), (**b**) **PtTThPor** (light), (**c**) **PdTThPor** (dark) and (**d**) **PdTThPor** (light), using a white-light LED source (irradiance of 50 mW cm^−2^ and a total light dosage of 270 J cm^−2^) for 90 min. C-: negative control; EC: experiment control. SSB: single-strand breakage of DNA. FPG SS: Formamido-pyrimidine DNA Glycosylase Sensitive Sites. ENDO III SS: Endonuclease III sensitive sites. Mean and standard deviation of three independent experiments. ** *p* = 0.0033; *** *p* = 0.0005; **** *p* < 0.0001.

On the other hand, **PdTThPor** porphyrin caused significant DNA damage proportional to the increase in its concentration when exposed to white light conditions, and the genotoxicity of the Pd(II) compound remained in the dark (Figure 7a,b). The experimental control showed considerably more breaks per 1000 base pairs (kbp) when compared to the negative control, as well as the three concentrations of porphyrin when exposed in the dark (*p* < 0.0001). The **PtTThPor** induced significant DNA damage at the two highest concentrations (10 µM: *p* = 0.0005 and 20 µM: *p* < 0.0001), which was also maintained in the dark with DNA in solution with 20 µM of porphyrin (*p* = 0.0033) (Figure 7c,d). For all treatments, most of the damage identified was in purines, since the Fpg enzyme showed more activity in all tests.

## 3. Materials and Methods

### 3.1. General

All chemical reagents were of analytical grade and purchased from Sigma-Aldrich^®^ (Burlington, MA, USA) and Oakwood Chemical^®^ (Estill, SC, USA) without any further purification. The calf-thymus acid desoxyribonucleic (CT-DNA) was lyophilized powder (Sigma-Aldrich^®^, São Paulo, Brazil, purity ≥ 99%). The concentration of the stock solutions of DNA was confirmed by UV-Vis analysis through the Beer-Lambert equation with the molar absorptivity (ε) value of 6600 M^−1^ cm^−1^ for CT-DNA at 260 nm (per nucleic acid) in Tris-HCl buffer (pH 7.4) solution and the water used in all experiments was milliQ grade.

### 3.2. Photobiological Parameters of Porphyrins

Stability, photo-stability, aggregation, ROS generation (by spectroscopy and EPR analysis) and partition coefficients of porphyrins **TThPor**, **PdTThPor** and **PtTThPor** were previously described by Tisoco and co-workers [19].

### 3.3. DNA Interactive Studies

UV-Vis absorption analysis for each porphyrin without and in the presence of successive additions of CT-DNA solution was obtained at 298.15 K in a DMSO(5%)/Tris-HCl pH 7.4 mixture buffered solution in the 250 to 800 nm range. The porphyrin concentration was fixed at 5.0 μM and CT-DNA was in the 0 to 50 μM range. The hyperchromicity (*H*%), red-shift (Δλ), binding constant (*K*_b_) and Gibb’s free-energy (ΔG°) values of the porphyrins **TThPor**, **PdTThPor** and **PtTThPor** were calculated according to the literature through Benesi-Hildebrand and free-energy equations [31].

Competitive binding assays between CT-DNA:dyes and thienyl-porphyrins by steady-state fluorescence emission analysis are recorded and the porphyrins **TThPor**, **PdTThPor** and **PtTThPor** in DMSO (5%)/Tris-HCl pH 7.4 mixture buffered solution (0 to 100 μM) were gradually added in a fixed concentration of ethidium bromide (EB; general intercalator; 10 μM; λ_exc_ = 510 nm, λ_em_ = 550–800 nm), acridine orange (AO; A-T rich intercalator; 10 μM; λ_exc_ = 490 nm, λ_em_ = 500–800 nm), 4′,6-diamidino-2-phenylindole (DAPI; minor groove binder; 10 μM; λ_exc_ = 359 nm, λ_em_ = 380–700 nm), methyl green (MG, major groove binder; 10 μM, λ_exc_ = 318 nm, λ_em_ = 330–600 nm) and CT-DNA (10 μM) in DMSO(5%)/Tris-HCl pH 7.4 mixture buffered solution. The DNA:dye adducts were incubated for 5 min after porphyrin addition for each measurement. The Stern-Volmer quenching (*K*_SV_) and bimolecular quenching rate (*k*_q_) constants of derivatives were calculated according to the DNA:dye fluorescence quenching using a plot of F_0_/F versus [porphyrin] and a ratio of *K*_SV_/τ_0_, where the τ_0_ denotes the fluorescence lifetime of DNA:dye (EB = 23.0 ns; AO = 2.20 ns; DAPI = 1.70 ns; MG = 2.80 ns), respectively. Binding (*K*_b_) constant and free-energy interaction (ΔG°) values are obtained by modifying Stern-Volmer and Gibb’s equation according to the literature [40].

Viscosity analyses were carried out using an Ostwald viscometer immersed in a water bath maintained at 298.15 K, according to the literature [41]. The CT-DNA concentration was kept constant in all experiments, while the porphyrin concentration was increased in the DMSO(5%)/Tris-HCl pH 7.4 mixture buffered solution. The flow time was measured at least three times with a digital stopwatch (Casio^®^), and the mean value was calculated. Data are presented as (η/η^0^)^1/3^ versus the ratio [porphyrin]/[CT-DNA], where η and η^0^ are the specific viscosities of CT-DNA in the presence and absence of the porphyrins **TThPor**, **PdTThPor** and **PtTThPor**, respectively.

Also, the circular dichroism (CD) spectra of the DNA solutions and in the presence of porphyrins **TThPor**, **PdTThPor** and **PtTThPor** were recorded in a Jasco spectropolarimeter, model J810-150S, at 298.15 K. The experiment was carried out starting with a solution of DNA dissolved in 50 mM Tris-HCl buffer (pH = 7.4) with 1.0% DMSO. The CD spectrum of this solution was recorded, and then aliquots of the solutions of each porphyrin were added in a concentration range between 11 and 55 μM with the same solvent, recording the spectrum after each addition.

### 3.4. Molecular Docking Procedure with DNA

The crystallographic structure of the DNA was obtained from the Protein Data Bank (PDB) with access code 1BNA [37]. The chemical structure of the porphyrins **TThP**, **PdTThPor** and **PtTThP** was built and minimized in terms of energy by Density Functional Theory (DFT), available in the Spartan’18 software (Wavefunction, Inc., Irvine, CA, USA) [42]. Molecular docking calculations were performed with GOLD 5.7 software (Cambridge Crystallographic Data Centre, Cambridge, CB2 1EZ, UK) [43]. Hydrogen atoms were added to the DNA following tautomeric states and ionization data inferred by GOLD 5.7 software (version 2022.3, Cambridge, UK). In silico calculations were performed in a 10 Å radius around the two main possible binding sites (major and minor grooves) [44]. The standard ChemPLP was used as a scoring function due to the best results obtained in previous work for porphyrins [45]. The figures for the best docking pose were generated with PyMOL Delano Scientific LLC software (Schrödinger, New York, NY, USA) [45]. Additionally, this same software was also used to detect the main interactions among **TThP**/**PdTThPor**/**PtTThPor** and nucleobases through a cut-off for the interaction of 4.2 Å [46] and an analysis of van der Waals radius superposition.

### 3.5. DNA Photo-Oxidation by UV-Vis Analysis

The photo-oxidation assays of DNA were conducted by absorption UV-Vis analysis at room temperature. Stock solutions of DNA (5.0 μM) were prepared in Tris-HCl buffer (pH 7.4) containing **TThP**, **PdTThPor** and **PtTThP** (at 5.0 μM) and the solutions were irradiated with a white-light LED source (irradiance of 50 mW cm^−2^ and a total light dosage of 90 J cm^−2^) in a time period of 30 min. The DNA was absorbed at 260 nm, and the plots of ln A_0_/A versus time for DNA gave a straight line from which the photo-oxidation rate (*k*_po_) constant was calculated.

### 3.6. Electrophoresis and Detection of DNA Oxidative Damage

The DNA template used for the detection and quantification of oxidative damage by porphyrins **TThP**, **PdTThPor** and **PtTThP** was the plasmid pCMUT, extracted from *Escherichia coli*. The plasmid was exposed to concentrations of 5.0 µM, 10 µM and 20 µM of the porphyrins, in the dark and in white-light LED conditions (irradiance of 50 mW cm^−2^ and a total light dosage of 270 J cm^−2^) for a period of 90 min using the DNA dosimeter system. After exposure, the DNA was incubated at 37 °C for 60 min with the enzymes formamido-pyrimidine DNA glycosylase (Fpg) and endonuclease III (Endo III), which recognize and cleave oxidized bases, mainly purines and pyrimidines, respectively.

In addition, the DNA was incubated without the presence of enzymes to detect single-stranded breakage (SSB). Then, the solutions were submitted to 0.8% agarose gel electrophoresis. The documentation of this gel was performed using the photo-documenter Amersham Imager 600 (General Electronic). In the gel, we are able to see if the exposure to the porphyrins under different conditions caused damage to DNA. When there is no DNA damage, a lower band is formed in the gel (FI), since the plasmid will stay in its supercoiled form. In the case of DNA damage, a second upper band is formed since the DNA is in a more relaxed form due to the enzyme’s activity.

Damage quantification was performed by band densitometry using the Image Quant 300 program (GE Healthcare, USA) [47]. Statistical analysis was performed using the GraphPad Prism program using the One-Way ANOVA test followed by Sidak, with a confidence interval of 95% (*p* < 0.05).

## 4. Conclusions

In summary, these results indicate that the thienyl-porphyrins with Pd(II) or Pt(II) polypyridyl complexes are promising dyes for DNA interaction. The insertion of the peripherally coordinated Pt(II) or Pd(II) complexes resulted in increased interaction with nucleic acids when compared to the non-cationic free-base porphyrin **TThPor**. These derivatives interact probably via secondary interactions via minor grooves around the DNA nucleobases, showing good binding parameters determined by absorption and emission analysis and molecular docking calculations. Additionally, Pd(II) or Pt(II) complexes (**PdTThPor** and **PtTThPor**) were able to promote photo-damage in the DNA, thus evidencing the main purpose of this work, which was to demonstrate the potential interaction and damage induction of these porphyrins in DNA. These findings may open up many opportunities for further studies on PDT treatments and their mechanisms of action.

## Figures and Tables

**Table 2:** Molecular docking score values (dimensionless) for the interaction between DNA and the porphyrins under study at the corresponding binding site.

Compound	Minor Groove	Major Groove
**TThPor**	69.0	44.5
**PdTThPor**	66.3	43.5
**PdTThPor**	62.3	42.2

**Table 3:** Photo-oxidation rate (*k*_po_) constants and half-life times in white-light LED conditions (irradiance of 50 mW cm^−2^ and a total light dosage of 90 J cm^−2^) for 30 min, using **TThPor**, **PdTThPor** and **PtTThPor**, in the presence of DNA, by absorption analysis at 298.15 K.

Porphyrin	*Q* (%) ^a^	*k*_po_ (min^−1^)	t_½_ (h)
**TThPor**	18.0	6.18 × 10^−3^ ± 0.03	1.87
**PdTThPor**	25.5	9.41 × 10^−3^ ± 0.05	1.23
**PtTThPor**	27.5	1.12 × 10^−2^ ± 0.04	1.03

^a^ Quenching (Q%) = A_0_ − A/A_0_ × 100%.

## Data Availability

All analyzed data are contained in the main text of the article. Raw data are available from the authors upon request.

## Supplementary Materials

The following supporting information can be downloaded at: https://www.mdpi.com/article/10.3390/molecules28135217/s1, Figure S1: UV-Vis spectra of the **TThPor** upon successive additions of CT-DNA concentrations (0 to 50 µM) in DMSO(5%)/Tris-HCl pH 7.4 mixture buffered solution. Graph plots of A_0_/(A – A_0_) versus 1/[CT-DNA]; Figure S2: Steady-state fluorescence emission spectra for EB:DNA without and in the presence of porphyrin **TThPor**, in DMSO(5%)/Tris-HCl pH 7.4 mixture buffered solution. Graphs plots shows the F_0_/F versus [porphyrin]; Figure S3: Steady-state fluorescence emission spectra for AO:DNA without and in the presence of porphyrin **TThPor**, in DMSO(5%)/Tris-HCl pH 7.4 mixture buffered solution. Graphs plots shows the F0/F versus [porphyrin]; Figure S4: Steady-state fluorescence emission spectra for DAPI:DNA without and in the presence of porphyrin **TThPor**, in DMSO(5%)/Tris-HCl pH 7.4 mixture buffered solution. Graphs plots shows the F_0_/F versus [porphyrin]; Figure S5: Steady-state fluorescence emission spectra for MG:DNA without and in the presence of porphyrin **TThPor**, in DMSO(5%)/Tris-HCl pH 7.4 mixture buffered solution. Graphs plots shows the F_0_/F versus [porphyrin]; Figure S6: Steady-state fluorescence emission spectra for EB:DNA without and in the presence of porphyrin **PdTThPor**, in DMSO(5%)/Tris-HCl pH 7.4 mixture buffered solution. Graphs plots shows the F0/F versus [porphyrin]; Figure S7: Steady-state fluorescence emission spectra for AO:DNA without and in the presence of porphyrin **PdTThPor**, in DMSO(5%)/Tris-HCl pH 7.4 mixture buffered solution. Graphs plots shows the F_0_/F versus [porphyrin]; Figure S8: Steady-state fluorescence emission spectra for DAPI:DNA without and in the presence of porphyrin **PdTThPor**, in DMSO(5%)/Tris-HCl pH 7.4 mixture buffered solution. Graphs plots shows the F_0_/F versus [porphyrin]; Figure S9: Steady-state fluorescence emission spectra for MG:DNA without and in the presence of porphyrin **PdTThPor**, in DMSO(5%)/Tris-HCl pH 7.4 mixture buffered solution. Graphs plots shows the F_0_/F versus [porphyrin]; Figure S10: CD spectra for DNA solution in Tris-HCl buffer (pH = 7.4, 1% DMSO) before and after successive additions of porphyrin **TThPor**; Figure S11: DNA photo-oxidation assay by UV-Vis analysis in DMSO(5%)/Tris-HCl pH 7.4 mixture buffered solution of porphyrin **TThPor;** Figure S12: DNA photo-oxidation assay by UV-Vis analysis in DMSO(5%)/Tris-HCl pH 7.4 mixture buffered solution of porphyrin **PdTThPor**; Figure S13: DNA photo-oxidation assay by UV-Vis analysis in DMSO(5%)/Tris-HCl pH 7.4 mixture buffered solution of porphyrin **PtTThPor**; Figure S14: Quantification of DNA lesions generated by porphyrins (a) **TThPor** (dark) and (b) **TThPor** (light), using white-light LED source (irradiance of 50 mW cm^−2^ and a total light dosage of 270 J cm^−2^) for 90 min. C-: negative control, EC: experiment control. SSB: single-strand breakage of DNA. FPG SS: Formamido-pyrimidine DNA Glycosylase Sensitive Sites. ENDO III SS: Endonuclease III sensitive sites. Mean and standard deviation of three independent experiments. ** *p* = 0.0033; *** *p* = 0.0005; **** *p* < 0.0001; Table S1: Molecular docking results for the interaction between DNA:TThPor, DNA:PtTThPor, and DNA:PdTThPor in the minor groove.

## Sample Availability

Samples of the compounds **TThPor**, **PdTThPo**r and **PtTThPor** are available from the authors.
