# Supplementary material for: DNA-Interactive and Damage Study with meso-Tetra(2-thienyl)porphyrins Coordinated with Polypyridyl Pd(II) and Pt(II) Complexes
Source: Molecules. 2023 Jul 5;28(13):5217. doi: 10.3390/molecules28135217 (PMC10343509; doi:10.3390/molecules28135217)
Supplement: Supplementary file 1 [file molecules-28-05217-s001.zip › molecules-2431969-supplementary.pdf]

Supplementary Information

DNA-Interactive and Damage Study with *meso*-Tetra(2-thienyl)porphyrins Coordinated with Polypyridyl Pd(II) and Pt(II) Complexes

Bernardo Almeida Iglesias [1,*], Níckolas Pippi Peranzoni [2], Sophia Iwersen Faria [2], Luana Belo Trentin [2], André Passaglia Schuch [2], Otávio Augusto Chaves [3], Renan Ribeiro Bertoloni [4], Sofia Nikolaou [4] and Kleber Thiago de Oliveira [5,*]

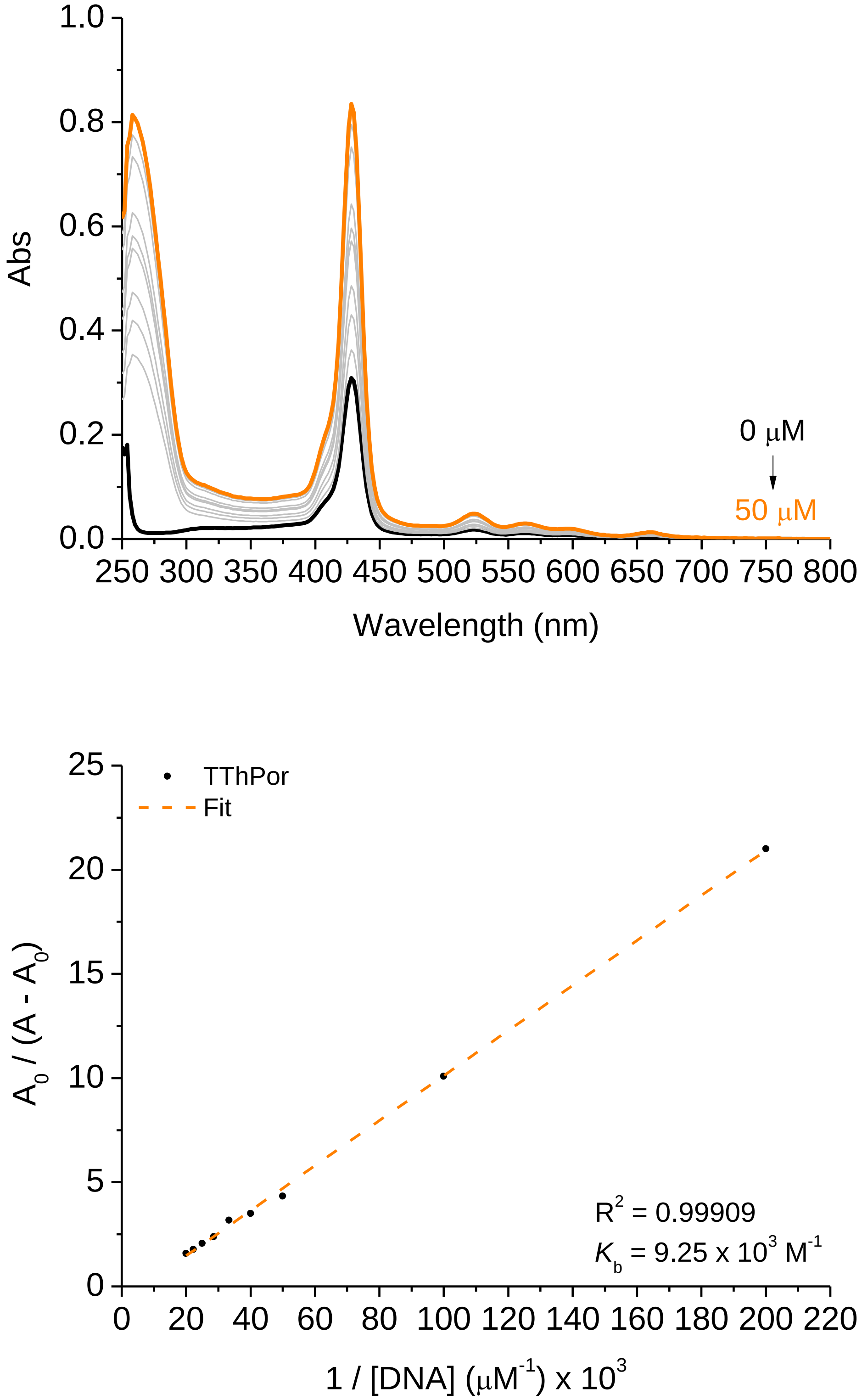

Figure S1. UV-Vis spectra of the **TThPor** upon successive additions of CT-DNA concentrations (0 to 50 μM) in DMSO(5%)/Tris-HCl pH 7.4 mixture buffered solution. Graph plots of $A_0/(A – A_0)$ versus 1/[CT-DNA].

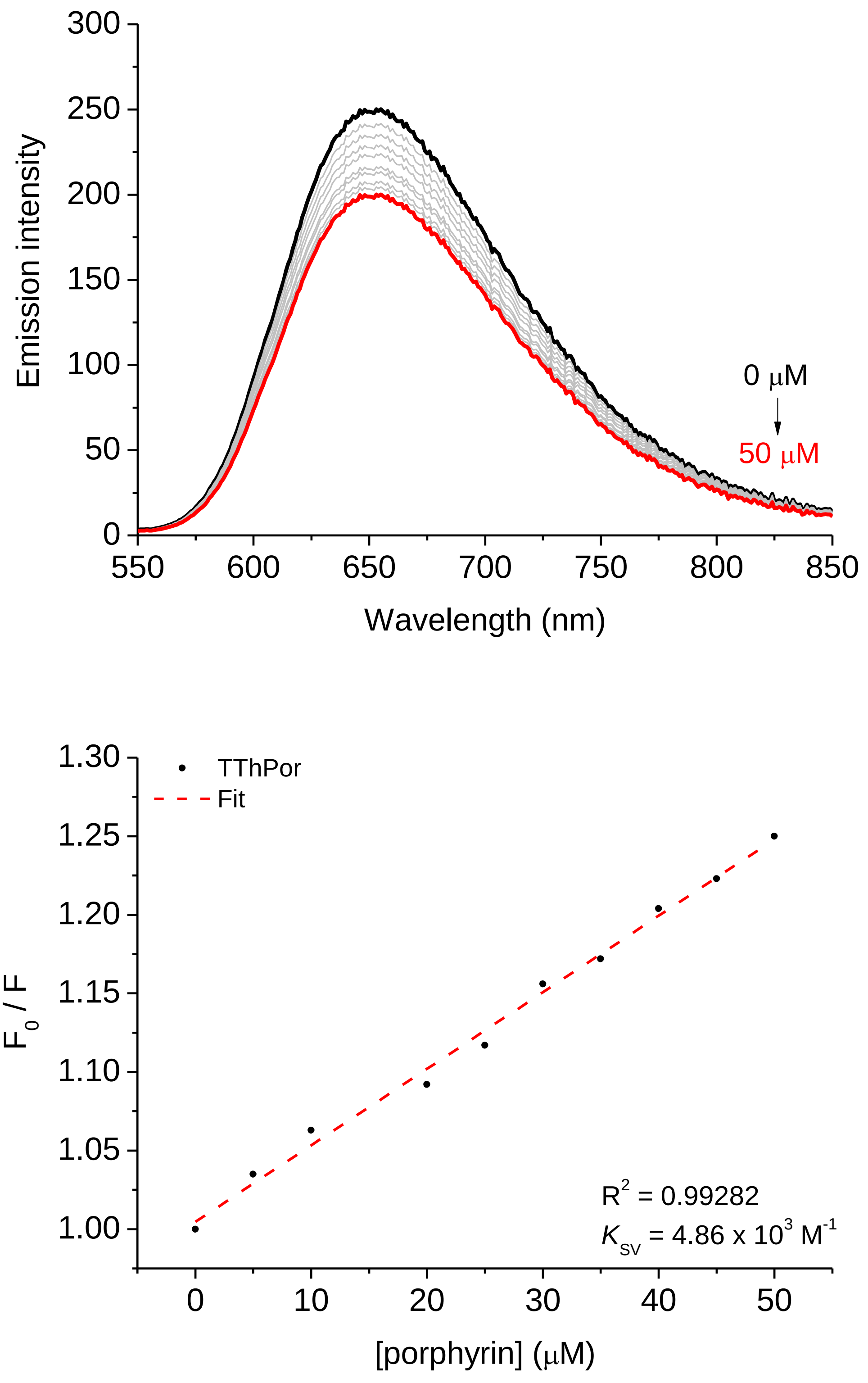

Figure S2. Steady-state fluorescence emission spectra for EB:DNA without and in the presence of porphyrin **TThPor**, in DMSO(5%)/Tris-HCl pH 7.4 mixture buffered solution. Graphs plots shows the F_0/F versus [porphyrin].

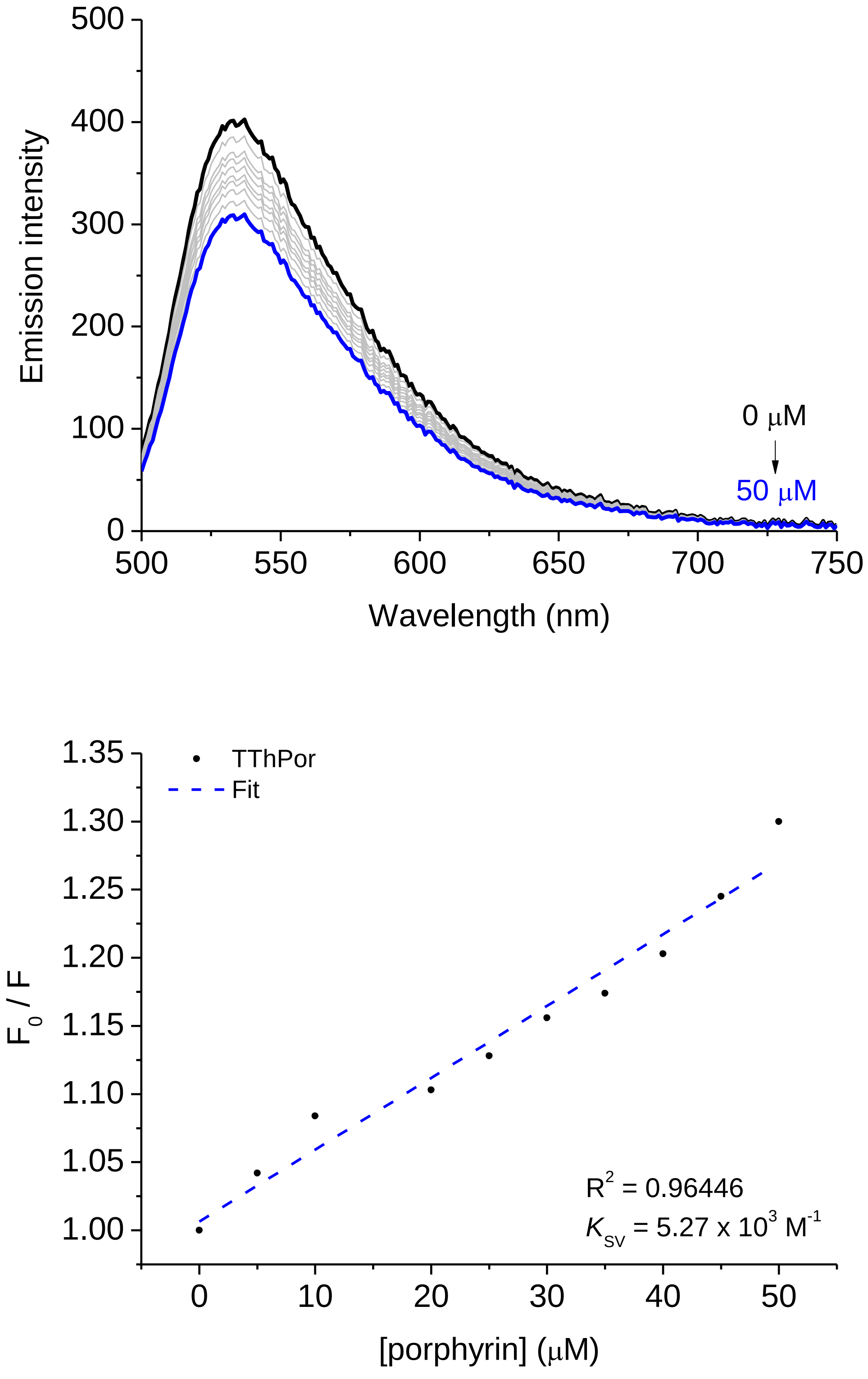

Figure S3. Steady-state fluorescence emission spectra for AO:DNA without and in the presence of porphyrin **TThPor**, in DMSO(5%)/Tris-HCl pH 7.4 mixture buffered solution. Graphs plots shows the F_0/F versus [porphyrin].

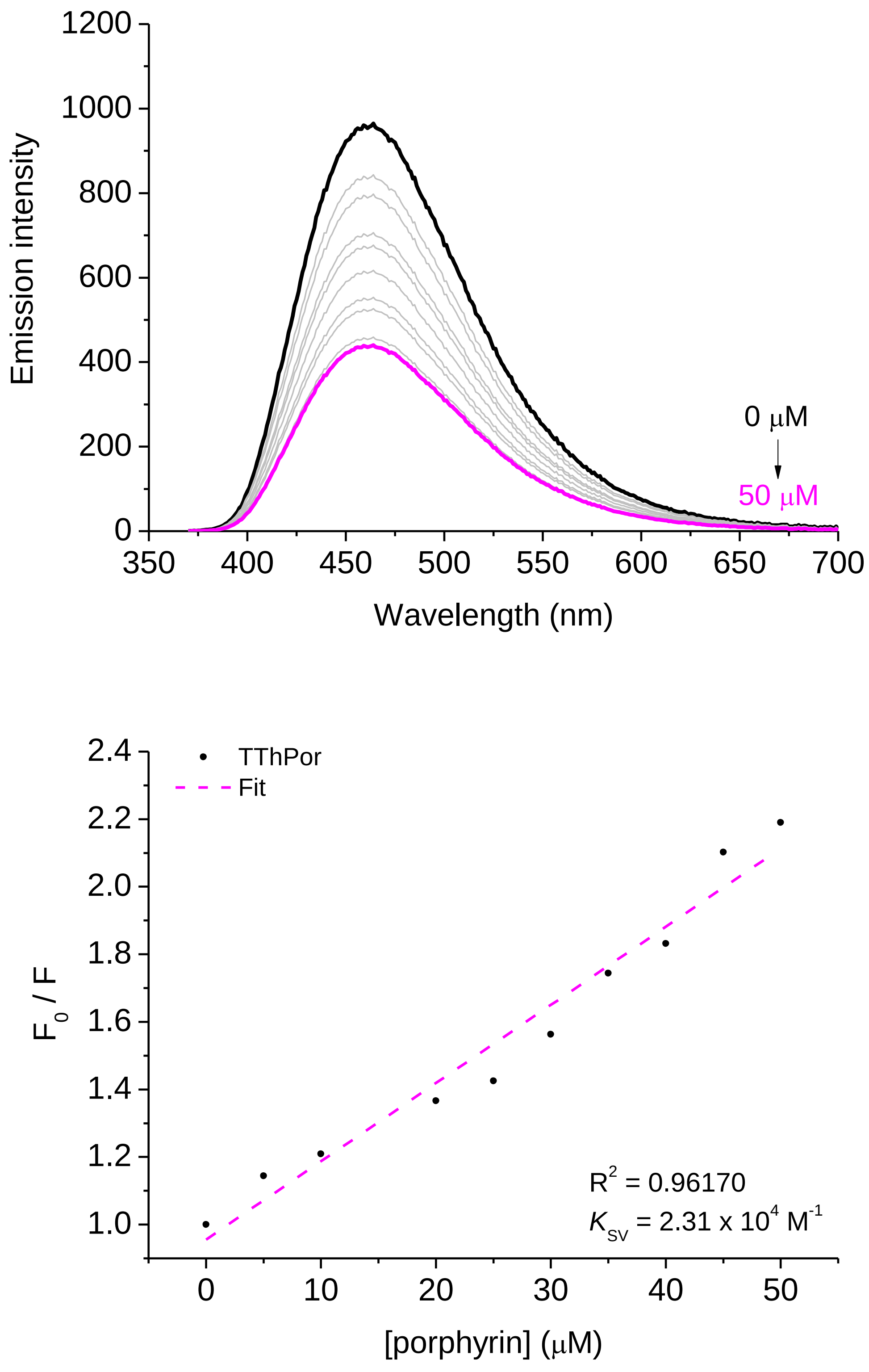

Figure S4. Steady-state fluorescence emission spectra for DAPI:DNA without and in the presence of porphyrin **TThPor**, in DMSO(5%)/Tris-HCl pH 7.4 mixture buffered solution. Graphs plots shows the F_0/F *versus* [porphyrin].

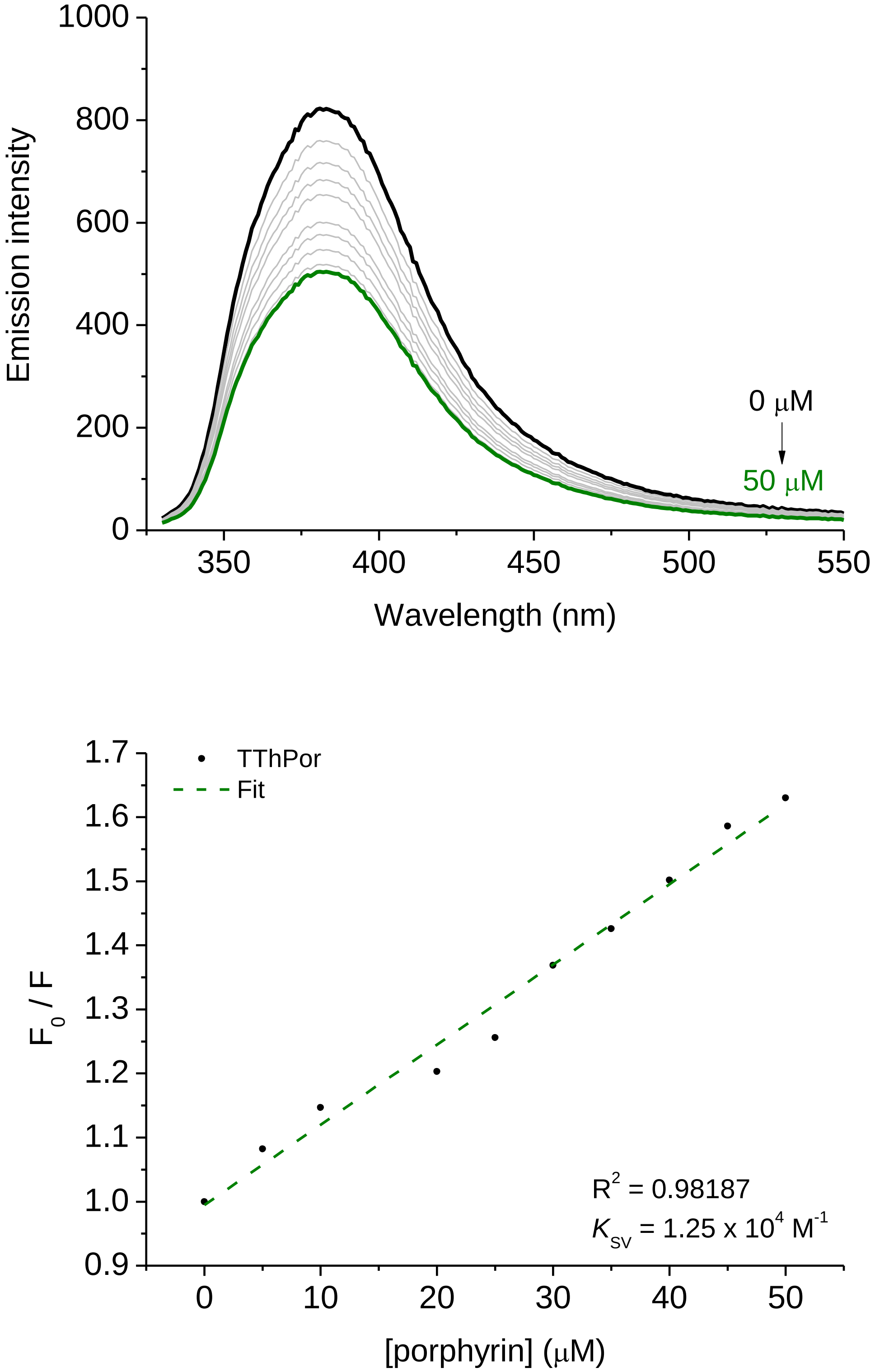

Figure S5. Steady-state fluorescence emission spectra for MG:DNA without and in the presence of porphyrin **TThPor**, in DMSO(5%)/Tris-HCl pH 7.4 mixture buffered solution. Graphs plots shows the F_0/F versus [porphyrin].

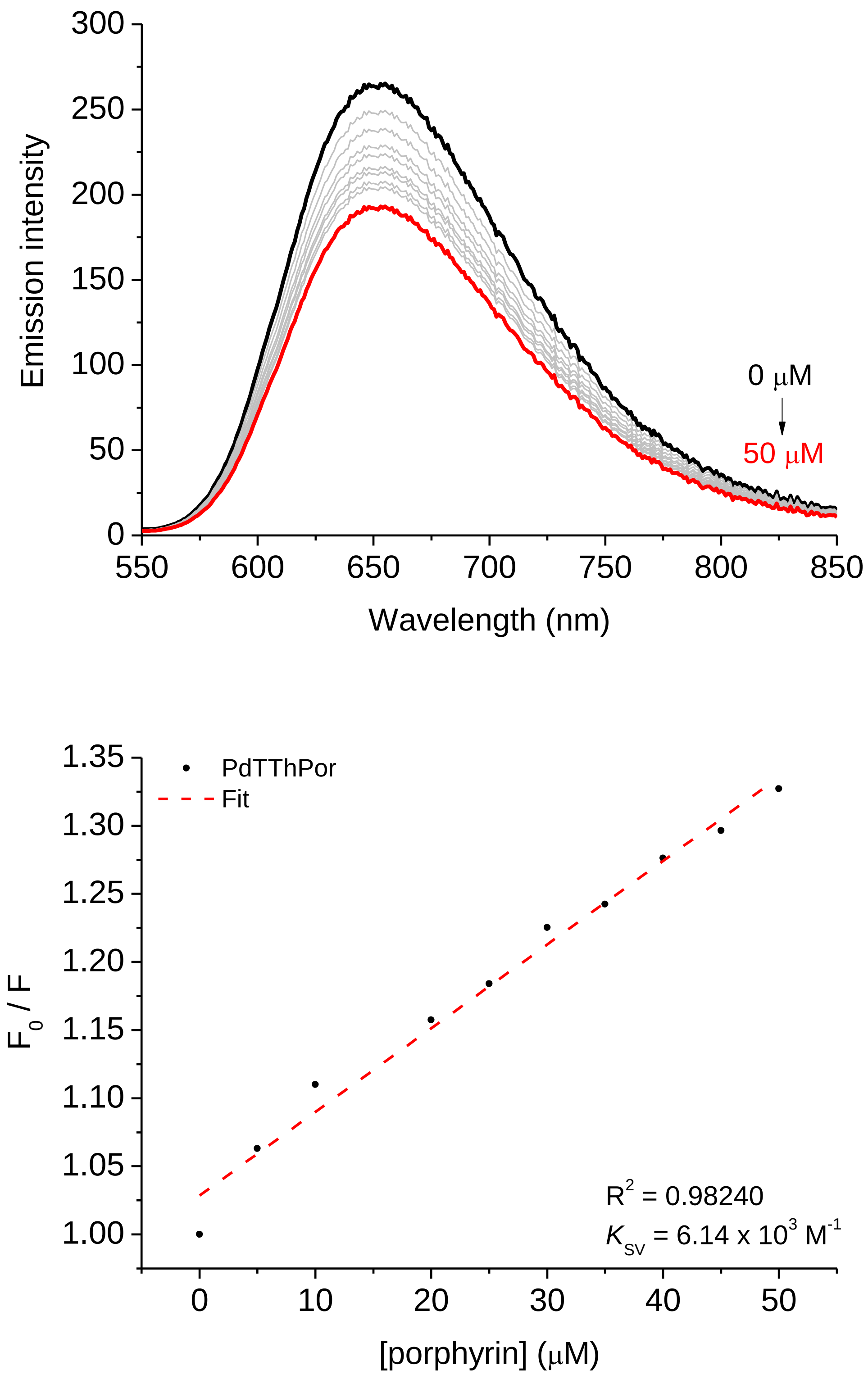

Figure S6. Steady-state fluorescence emission spectra for EB:DNA without and in the presence of porphyrin **PdTThPor**, in DMSO(5%)/Tris-HCl pH 7.4 mixture buffered solution. Graphs plots shows the F_0/F versus [porphyrin].

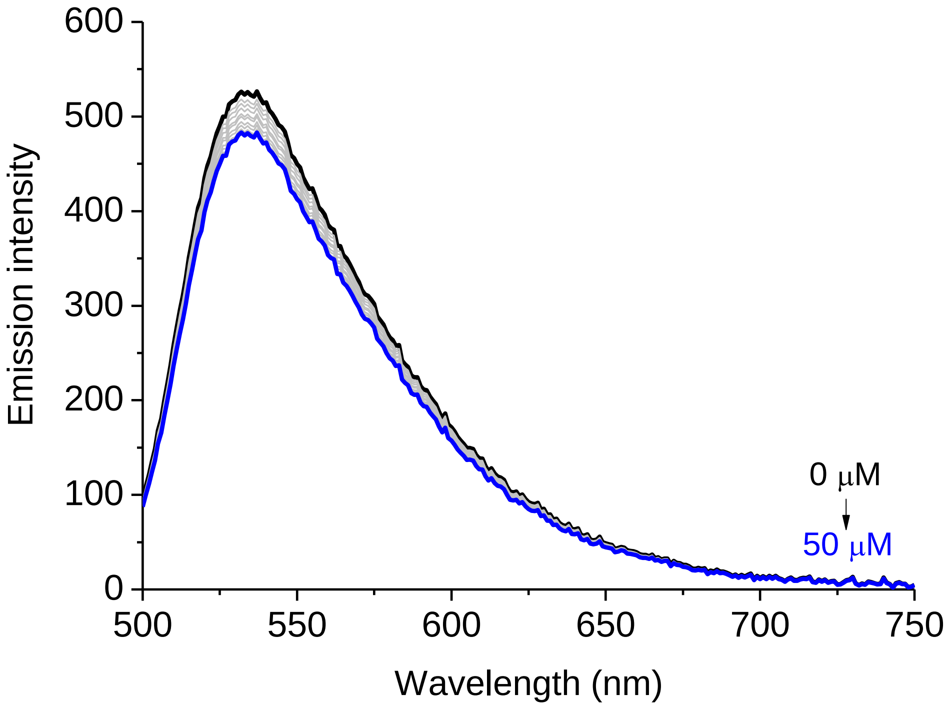

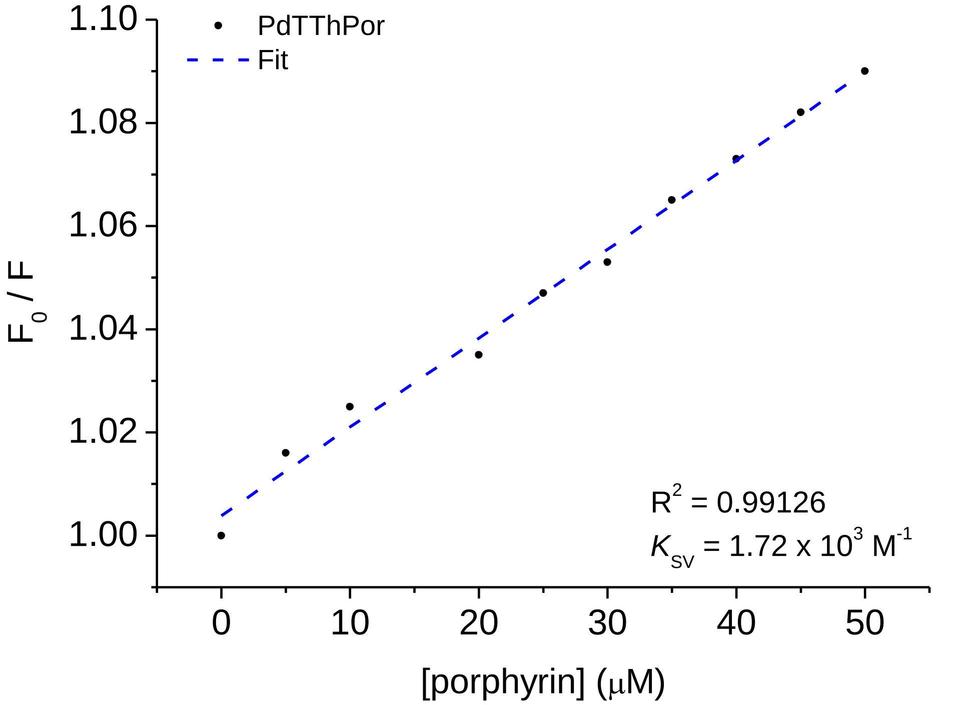

Figure S7. Steady-state fluorescence emission spectra for AO:DNA without and in the presence of porphyrin **PdTThPor**, in DMSO(5%)/Tris-HCl pH 7.4 mixture buffered solution. Graphs plots shows the F_0/F versus [porphyrin].

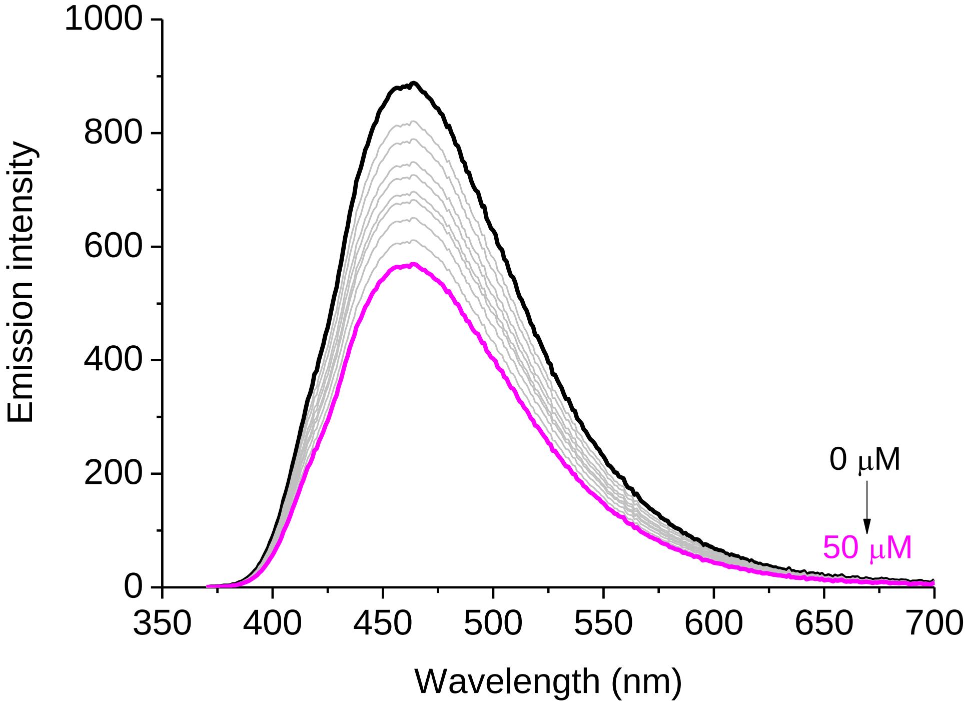

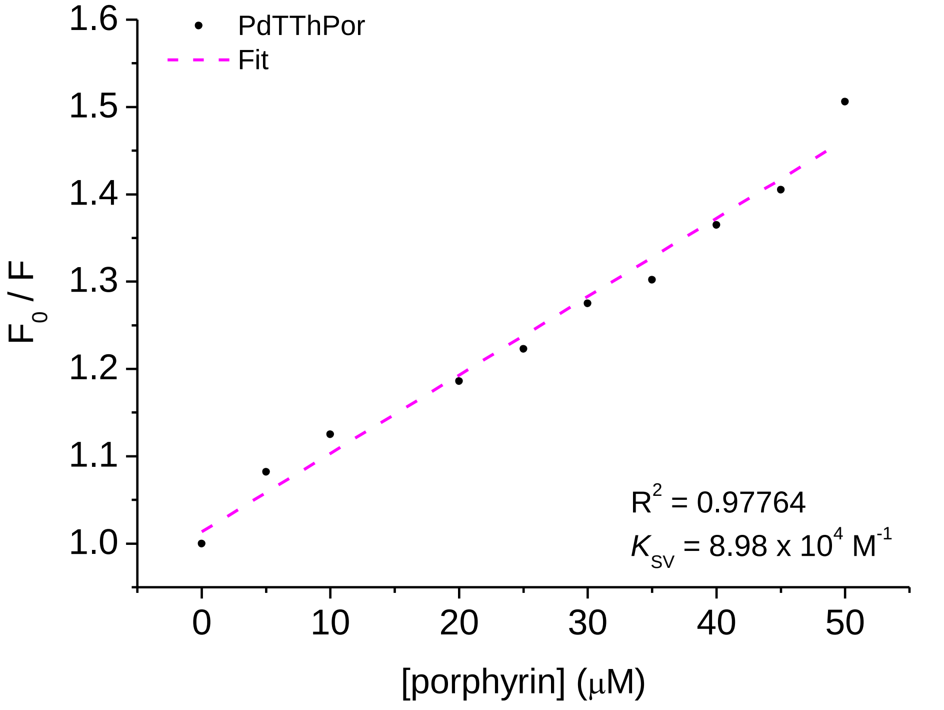

Figure S8. Steady-state fluorescence emission spectra for DAPI:DNA without and in the presence of porphyrin **PdTThPor**, in DMSO(5%)/Tris-HCl pH 7.4 mixture buffered solution. Graphs plots shows the F_0/F versus [porphyrin].

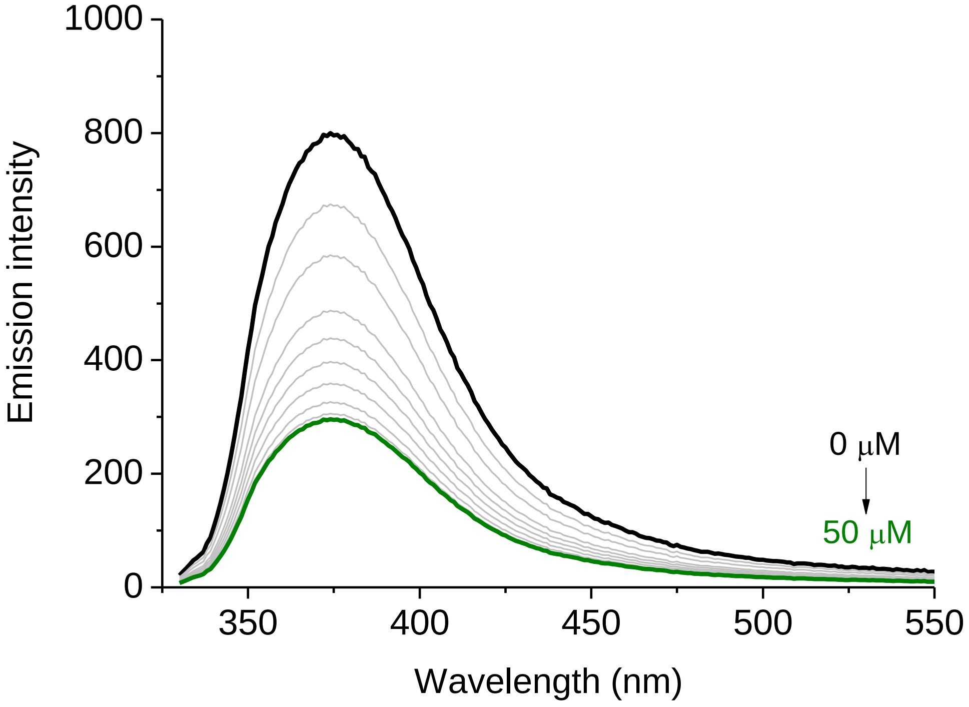

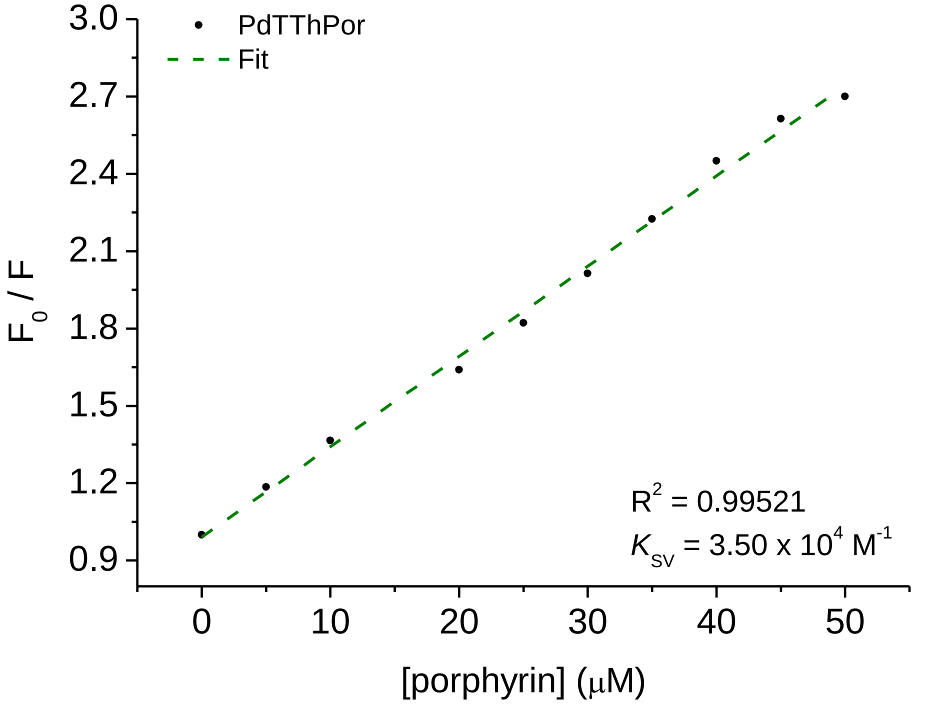

Figure S9. Steady-state fluorescence emission spectra for MG:DNA without and in the presence of porphyrin **PdTThPor**, in DMSO(5%)/Tris-HCl pH 7.4 mixture buffered solution. Graphs plots shows the F_0/F *versus* [porphyrin].

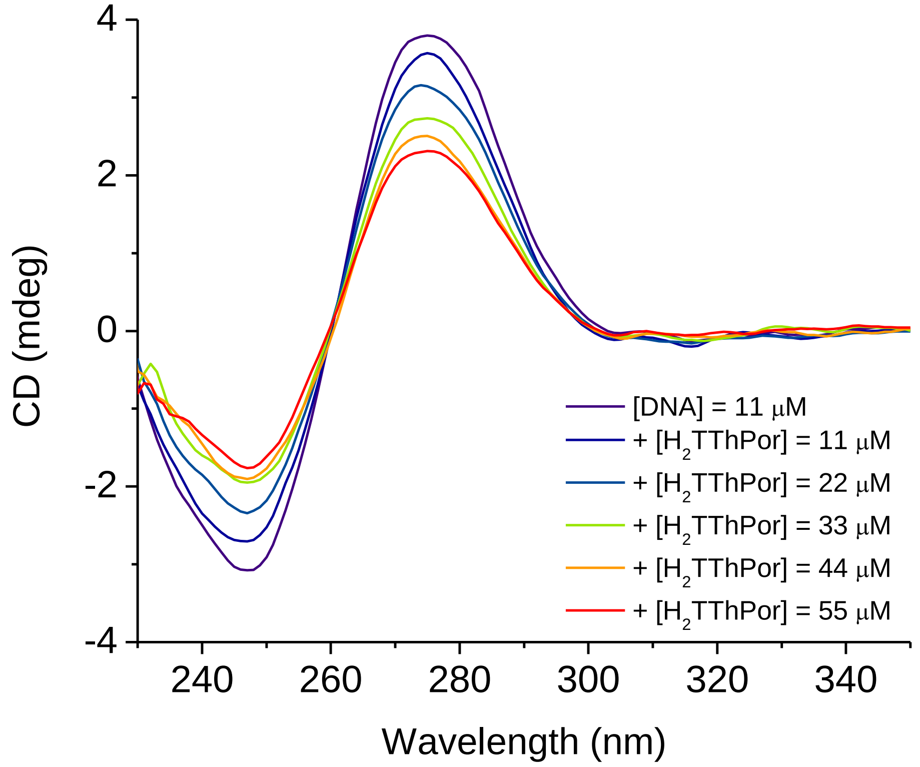

Figure S10. CD spectra for DNA solution in Tris-HCl buffer (pH = 7.4, 1% DMSO) before and after successive additions of porphyrin **TThPor**.

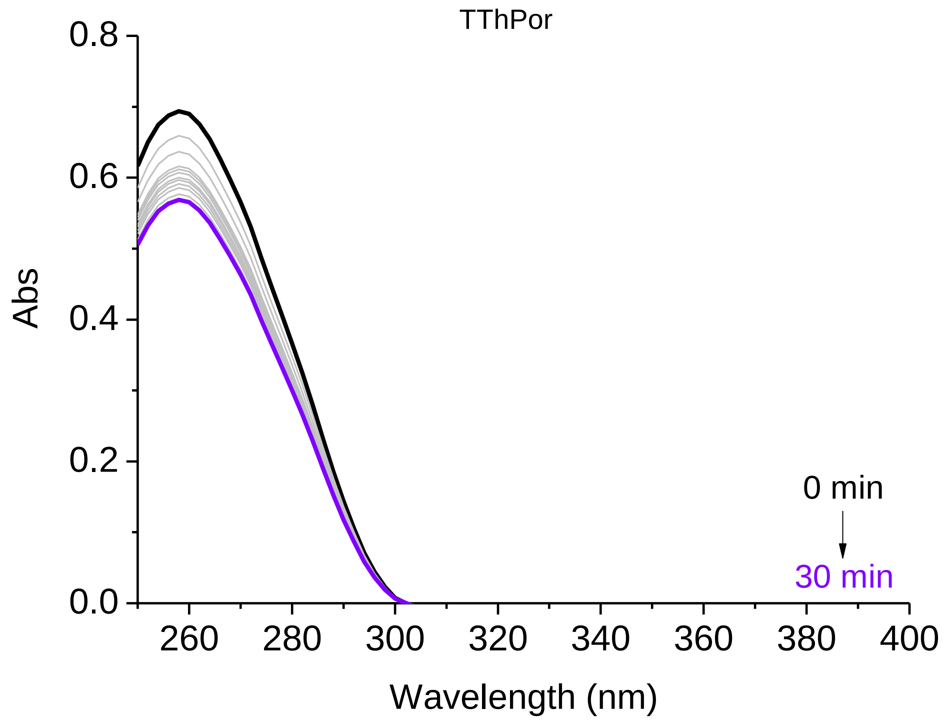

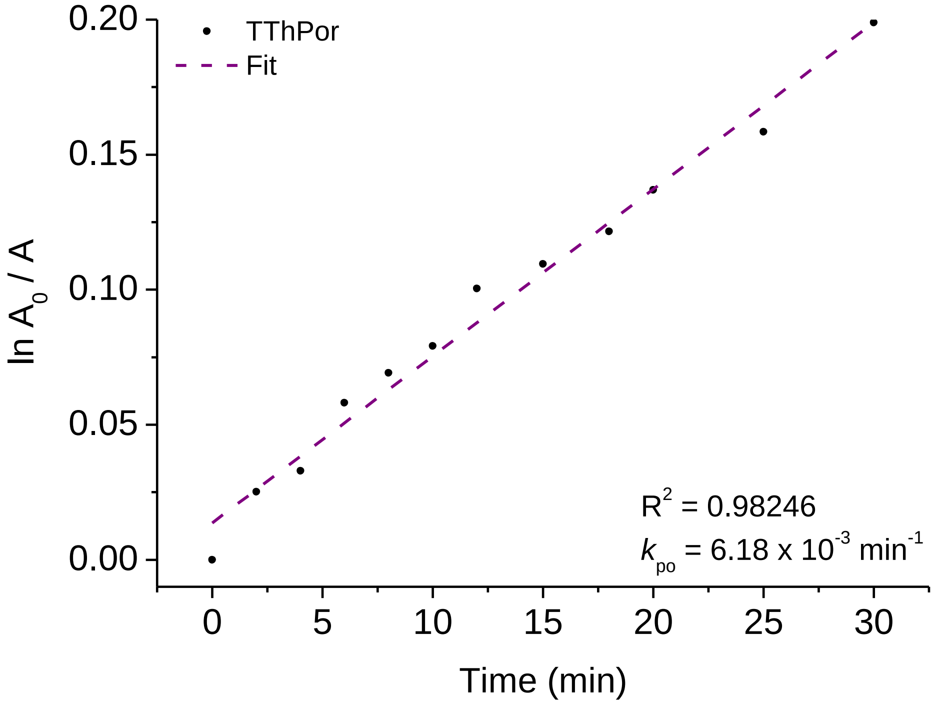

Figure S11. DNA photo-oxidation assay by UV-Vis analysis in DMSO(5%)/Tris-HCl pH 7.4 mixture buffered solution of porphyrin **TThPor**.

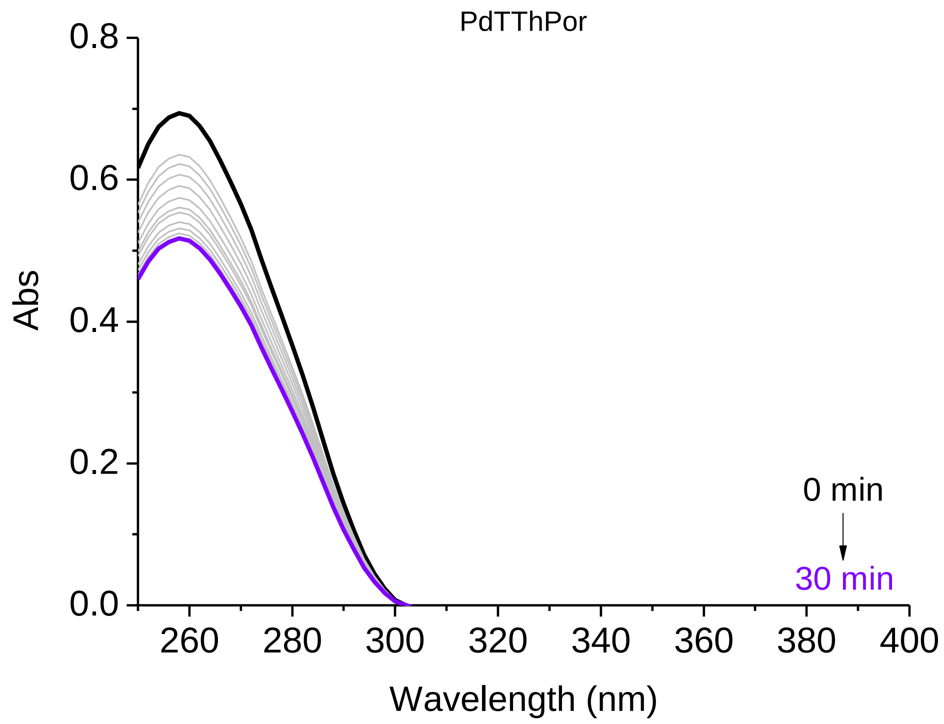

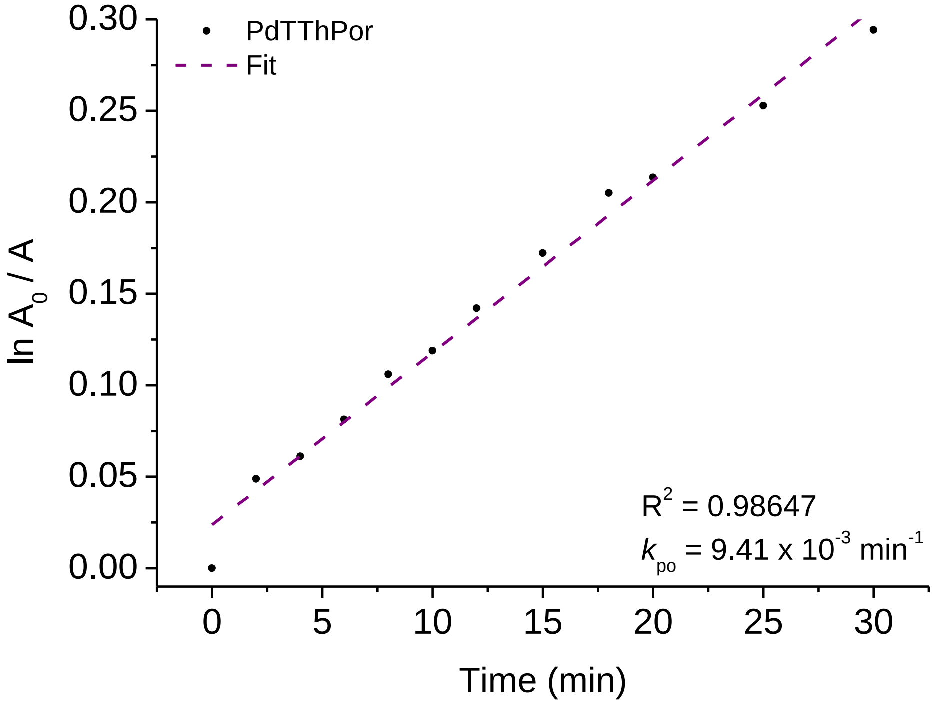

Figure S12. DNA photo-oxidation assay by UV-Vis analysis in DMSO(5%)/Tris-HCl pH 7.4 mixture buffered solution of porphyrin **PdTThPor**.

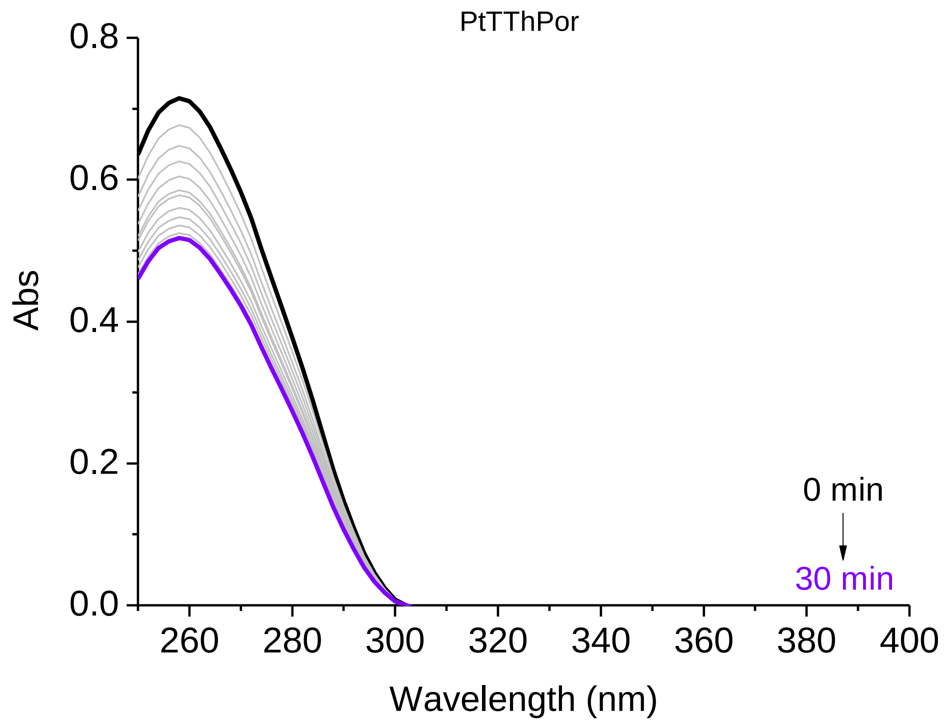

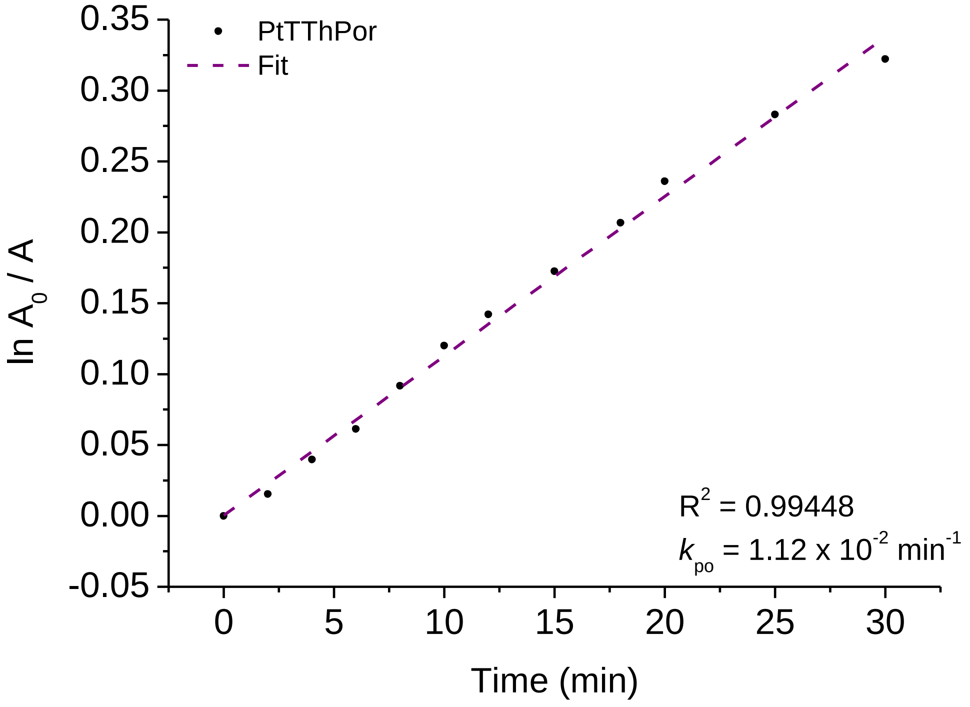

Figure S13. DNA photo-oxidation assay by UV-Vis analysis in DMSO(5%)/Tris-HCl pH 7.4 mixture buffered solution of porphyrin **PtTThPor**.

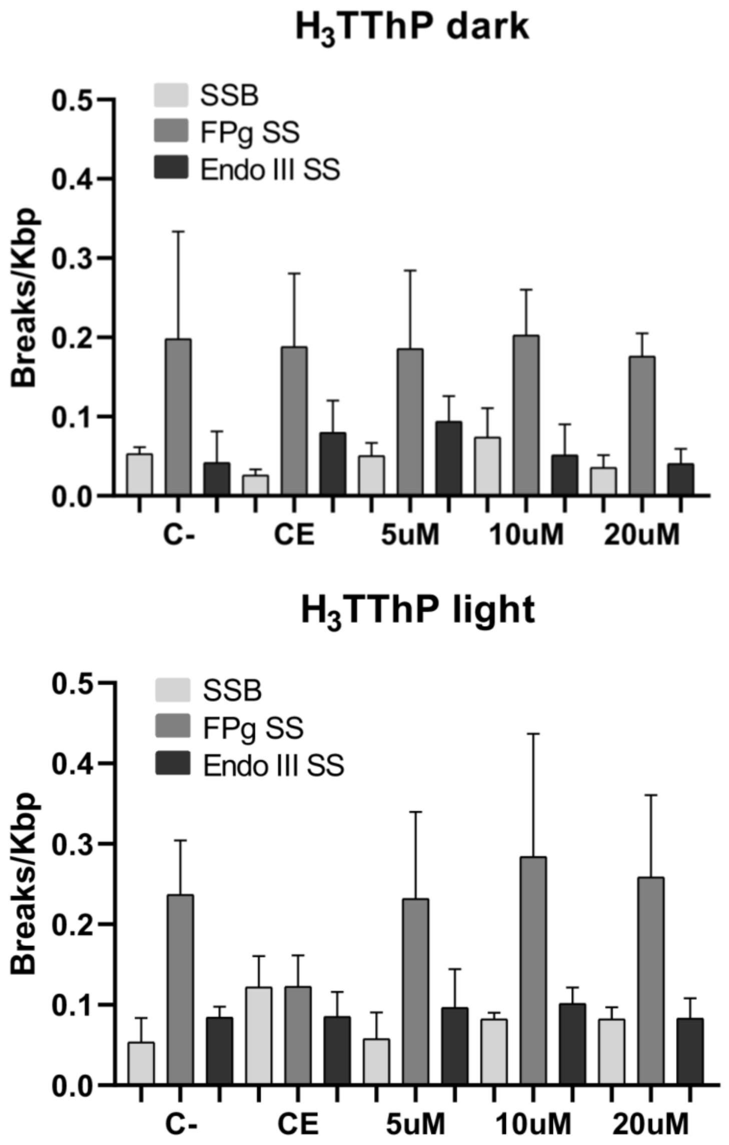

Figure S14. Quantification of DNA lesions generated by porphyrins (**a**) **TThPor** (dark) and (**b**) **TThPor** (light), using white-light LED source (irradiance of 50 mW cm^{-2} and a total light dosage of 270 J cm^{-2}) for 90 min. C-: negative control, EC: experiment control. SSB: single-strand breakage of DNA. FPG SS: Formamido-pyrimidine DNA Glycosylase Sensitive Sites. ENDO III SS: Endonuclease III sensitive sites. Mean and standard deviation of three independent experiments. ** $p = 0.0033$; *** $p = 0.0005$; **** $p < 0.0001$.

Table S1. Molecular docking results for the interaction between DNA:TThPor, DNA:PtTThPor, and DNA:PdTThPor in the minor groove.

Code	Nitrogenated bases	Interaction	Distance (Å)
**H_2TTP**	DA-05	Van der Waals	2.10
	DA-06	Van der Waals	2.70
	DT-07	Van der Waals	2.70
	DT-08	Van der Waals	2.80
	DA-18	Van der Waals	2.40
	DT-19	Van der Waals	2.40
	DT-20	Van der Waals	2.30
**PtbpyTTP**	DA-06	Van der Waals	3.20
	DT-19	Van der Waals	3.00
	Ribose-DT-20	Van der Waals	3.60
	Ribose-DC-21	Van der Waals	1.90
	Ribose-DG-22	Van der Waals	3.20
**PdbpyTTP**	DA-06	Van der Waals	2.30
	Ribose-DC-09	Van der Waals	3.30
	DT-19	Van der Waals	3.70
	Ribose-DT-20	Van der Waals	3.10
	DC-21	Van der Waals	2.30
	DG-22	Van der Waals	2.40
